# Cell salvage and donor blood transfusion during cesarean section: A pragmatic, multicentre randomised controlled trial (SALVO)

**DOI:** 10.1371/journal.pmed.1002471

**Published:** 2017-12-19

**Authors:** Khalid S. Khan, Philip A. S. Moore, Matthew J. Wilson, Richard Hooper, Shubha Allard, Ian Wrench, Lee Beresford, Tracy E. Roberts, Carol McLoughlin, James Geoghegan, Jane P. Daniels, Sue Catling, Vicki A. Clark, Paul Ayuk, Stephen Robson, Fang Gao-Smith, Matthew Hogg, Doris Lanz, Julie Dodds, on behalf of the SALVO study group

**Affiliations:** 1 Women’s Health Research Unit, Barts and The London School of Medicine and Dentistry, Queen Mary University of London, United Kingdom; 2 Birmingham Women’s Hospital, Birmingham, United Kingdom; 3 School of Health and Related Research, University of Sheffield, United Kingdom; 4 Pragmatic Clinical Trials Unit, Centre for Primary Care and Public Health, Queen Mary University of London, United Kingdom; 5 NHS Blood and Transplant, London, United Kingdom; 6 Sheffield Teaching Hospitals NHS Foundation Trust, Sheffield, United Kingdom; 7 Health Economics Unit, Institute of Applied Health Research, University of Birmingham, United Kingdom; 8 Nottingham Clinical Trials Unit, University of Nottingham, United Kingdom; 9 Singleton Hospital, Swansea, United Kingdom; 10 Simpson Centre for Reproductive Health, Royal Infirmary of Edinburgh, Edinburgh, United Kingdom; 11 Royal Victoria Infirmary, Newcastle-upon-Tyne, United Kingdom; 12 Institute of Cellular Medicine, Newcastle University, United Kingdom; 13 Peri-operative, Critical Care and Trauma Trials Group, University of Birmingham, United Kingdom; 14 Royal London Hospital, Barts Health NHS Trust, London, United Kingdom; University of Manchester, UNITED KINGDOM

## Abstract

**Background:**

Excessive haemorrhage at cesarean section requires donor (allogeneic) blood transfusion. Cell salvage may reduce this requirement.

**Methods and findings:**

We conducted a pragmatic randomised controlled trial (at 26 obstetric units; participants recruited from 4 June 2013 to 17 April 2016) of routine cell salvage use (intervention) versus current standard of care without routine salvage use (control) in cesarean section among women at risk of haemorrhage. Randomisation was stratified, using random permuted blocks of variable sizes. In an intention-to-treat analysis, we used multivariable models, adjusting for stratification variables and prognostic factors identified a priori, to compare rates of donor blood transfusion (primary outcome) and fetomaternal haemorrhage ≥2 ml in RhD-negative women with RhD-positive babies (a secondary outcome) between groups. Among 3,028 women randomised (2,990 analysed), 95.6% of 1,498 assigned to intervention had cell salvage deployed (50.8% had salvaged blood returned; mean 259.9 ml) versus 3.9% of 1,492 assigned to control. Donor blood transfusion rate was 3.5% in the control group versus 2.5% in the intervention group (adjusted odds ratio [OR] 0.65, 95% confidence interval [CI] 0.42 to 1.01, *p* = 0.056; adjusted risk difference −1.03, 95% CI −2.13 to 0.06). In a planned subgroup analysis, the transfusion rate was 4.6% in women assigned to control versus 3.0% in the intervention group among emergency cesareans (adjusted OR 0.58, 95% CI 0.34 to 0.99), whereas it was 2.2% versus 1.8% among elective cesareans (adjusted OR 0.83, 95% CI 0.38 to 1.83) (interaction *p* = 0.46). No case of amniotic fluid embolism was observed. The rate of fetomaternal haemorrhage was higher with the intervention (10.5% in the control group versus 25.6% in the intervention group, adjusted OR 5.63, 95% CI 1.43 to 22.14, *p* = 0.013). We are unable to comment on long-term antibody sensitisation effects.

**Conclusions:**

The overall reduction observed in donor blood transfusion associated with the routine use of cell salvage during cesarean section was not statistically significant.

**Trial registration:**

This trial was prospectively registered on ISRCTN as trial number 66118656 and can be viewed on http://www.isrctn.com/ISRCTN66118656.

## Abstract 2

In a multi-center randomized trial, Khalid Khan and colleagues examine the effect of routine use of cell salvage during cesarean section vs. standard care on the donor blood transfusion rates.

## Author summary

**Why was this study done?:**

- Given that cesarean section rates are rising worldwide, the need for promoting alternatives to blood transfusion in cesareans, such as harnessing the patient’s own reserves where feasible, is well recognised.
- While cell salvage in operations outside of obstetrics has been shown to reduce the need for donor blood transfusion, its effectiveness in cesarean section is unclear and is based on only two small controlled trials of cell salvage in cesarean section, with imprecise and inconclusive findings.
- Current obstetric guidelines on cell salvage have focused attention on the lack of high-quality research, recognising that although there is potentially a place for cell salvage in emergency blood loss during cesarean section, its use remains controversial.

**What did the researchers do and find?:**

- We conducted a multicentre randomised controlled trial, including 3,028 women at risk of haemorrhage during cesarean section, to test the effect of the routine use of cell salvage compared to the current standard of care on the need for donor blood transfusion.
- While donor blood transfusion rates were lower in the cell salvage group than in the control group (2.5% versus 3.5%, meaning that, on average, 1 in every 100 mothers given cell salvage avoided a donor blood transfusion), the difference between the groups was not statistically significant.
- Additionally, the study showed that in women with RhD-negative blood type who gave birth to RhD-positive babies, cell salvage was associated with increased maternal exposure to fetal blood.

**What do these findings mean?:**

- These findings indicate that routine cell salvage does not lead to a statistically significant reduction in donor blood transfusion rates in all women at risk of haemorrhage during cesarean section.

## Introduction

Childbirth by cesarean section is on the rise worldwide [1]. Excessive blood loss (haemorrhage) is an important cause of maternal death [2], emergency hysterectomy [3], and maternal critical care admission [4] among women undergoing a cesarean birth [5]. The treatment of major haemorrhage, in addition to optimising red cell mass and managing anaemia, includes strategies to minimise blood loss. Donor (allogeneic) blood transfusion is employed when the operative loss is life-threatening or when the mother has severe anaemia following arrest of haemorrhage. Red cell concentrates used in donor transfusion are a finite, nationally pooled resource in demand simultaneously by many clinical services [6]. Such transfusions also carry risks for recipients [7]. To promote alternatives to donor transfusion, harnessing the patient’s own reserves where feasible, is a recognised need [8,9].

Along with surgical expedience and medical therapy (including tranexamic acid [10,11]), the use of intraoperative cell salvage may reduce the pressure on transfusion services. Cell salvage, which collects, processes, and returns the woman’s own blood lost during surgery, is increasingly being deployed during cesareans. In theory, it reduces the infectious and allergenic risks associated with donor blood transfusion. It has also been shown to reduce the need for such transfusions in a wide spectrum of surgical disciplines [12,13]. However, obstetric practitioners remain concerned about the risk of amniotic fluid embolism and red cell isoimmunisation with the use of cell salvage [14,15]. Evidence for its effective, safe use in obstetrics is limited [16–18], and our systematic review [16] identified only 2 small randomised controlled trials with inconclusive findings [19,20]; thus, opinion about its value is not yet solidified [18].

We conducted a large, pragmatic, multicentre randomised trial to determine whether the routine use of cell salvage during cesarean section in women at risk of haemorrhage could safely reduce the need for donor blood transfusion in comparison to the current standard of care, where salvage is not routinely used.

## Methods

### Study design and setting

The SALVO study was designed as a pragmatic, multicentre individually randomised controlled trial with cost-effectiveness analysis. The study protocol was approved by the UK National Research Ethics Committee (North West–Haydock, approval number 12/NW/0513), and local permission was obtained in all participating obstetric units. The study protocol is available as S1 Text and can also be accessed at https://njl-admin.nihr.ac.uk/document/download/2007068. The trial was conducted in 26 UK obstetric units. No changes to the protocol design, statistical parameters, outcomes, eligibility criteria, or intervention were introduced during the study. Three substantial amendments to the protocol concerned changes to recruitment materials and strategies as well as clarifications. The findings are reported as per CONSORT guidelines (S2 Text).

### Participants

Our sample consisted of women who were admitted to the labour ward for delivery by emergency or elective cesarean section, with an identifiable increased risk of haemorrhage, who were at least 16 years of age, and able to understand written and spoken English for informed consent. We defined increased risk of haemorrhage as any emergency cesarean or as an elective cesarean for any reason other than maternal preference or known breech presentation, i.e., we excluded women undergoing an elective first cesarean due to either maternal preference or known breech presentation. We also excluded women with contraindications to either cell salvage or donor blood transfusion, such as active malignancy; sickle cell disease or trait; cultural, religious, or social beliefs against donor blood transfusion; or rare antibodies restricting the use of cross-matched donor blood.

All study participants were provided with antenatal information about the study, and gave informed consent before being enrolled. All participants admitted for elective cesarean section gave written informed consent before enrolment. In the case of participants undergoing emergency cesarean sections, either written consent was obtained before enrolment, or, if this was not possible due to the urgency of the operation, verbal consent was obtained before enrolment, and written informed consent was then sought after delivery.

### Randomisation and masking

Participating women were randomised by entry into a bespoke online system, using random permuted blocks of variable sizes to maintain allocation concealment, to either intervention or control, at a ratio of 1:1. Randomisation was stratified by treatment centre, indication for cesarean (emergency versus elective), placentation (abnormal versus normal), and multiple birth (twins or more versus singleton). Classification of indication for cesarean was based on urgency of delivery [21,22] as follows: emergency cesareans had varying levels of urgency based on the threat or potential threat to the life of the woman or fetus, whereas elective cesareans had no maternal or fetal compromise. Abnormal placentation was defined as a pathologically low-lying placenta (placenta praevia) or abnormally invasive placenta (placenta accreta, increta, or percreta) [23].

Allocation concealment with third-party randomisation helped minimise selection bias. However, given the nature of the intervention, it was not possible to blind local treatment staff to the allocation post-randomisation, but in general the staff caring postpartum were different to those involved in intraoperative care. Performance bias as a result of knowing the participant’s allocation could lead transfusion rates to vary. Pragmatically, the need for donor blood transfusion postpartum was determined according to the policies of each participating hospital, and donor blood transfusion rates and transfusion thresholds were monitored for compliance with these.

### Procedures

Participants were allocated either to cesarean section with routine use of cell salvage (intervention group), i.e., salvage equipment set up at the outset of cesarean to collect, process, and return blood lost at surgery after delivery of baby, or to cesarean section with the usual standard of care (control group), i.e., without routine use of cell salvage. In life-threatening acute haemorrhage, women were managed in line with the standard of care for such an emergency [2,23], which potentially included the use of cell salvage in the control group.

The intervention was delivered by staff (anaesthetists, operating department practitioners, midwives, or nurses as per local policy) who had been formally trained in the use of the cell salvage equipment, in accordance with local procedures and requirements for competence. In line with the pragmatic nature of the trial, no specific cell saver model was prescribed, and both standard and continuous transfusion models were in use.

For patients randomised to the intervention, full cell saver set-up for both collection and processing was mandated as part of the study protocol, as was the return of any volume of processed blood. Other process factors, such as swab washing [24], leukocyte depletion filter use, or number of suckers used, were left to local policy—although swab washing was encouraged as it was expected to increase the volume of blood available for processing and thus for re-transfusion.

The above process factors and adherence or non-adherence to the allocated intervention were captured on case report forms. For participants allocated to the intervention, we documented whether non-adherence was due to technical failure of the equipment or whether cell salvage was not set up, in violation of the protocol. For participants allocated to the control group, we documented whether non-adherence was due to acute emergency blood loss or whether cell salvage was set up from the beginning of the procedure, in violation of the protocol. As part of the continuous central trial oversight, sites with high rates of deviation were contacted and encouraged to review their procedures and equipoise.

Participants were followed up until hospital discharge. Postnatal investigations captured the outcomes listed below. RhD-negative women with RhD-positive babies were assessed for anti-D dose given after delivery and exposure to fetal blood by a screening acid elution test (Kleihauer) to determine if additional anti-D was needed. Confirmatory flow cytometry tests were documented for Kleihauer tests indicating a fetomaternal haemorrhage of >2 ml. If additional anti-D was indicated or where fetomaternal haemorrhage was >4 ml, the results of repeat testing undertaken after 72 hours were documented to establish clearance of fetal cells from the maternal circulation [25]. Adverse events were monitored, investigated, classified (serious or not; related or not), and reported to capture data on the safety of cell salvage.

### Outcomes

The primary outcome was the rate of women receiving donor blood transfusion to manage haemorrhage and its consequences, either during cesarean section or between surgery and hospital discharge. The primary outcome was assessed at sites from medical records and subsequently verified by cross-checking transfusion laboratory records.

Secondary outcomes included units of blood transfused, time to first mobilisation, length of hospital stay, pre- and postoperative serum haemoglobin, fetomaternal haemorrhage measured by Kleihauer acid elution test, maternal fatigue captured using the Multidimensional Fatigue Inventory (MFI) [26], safety outcomes (including transfusion reactions), costs of resources and service provision, and process outcomes (including volume of salvaged blood returned and technical failure of cell salvage).

### Analysis

A sample size of 3,050 women (1,525 per group) was planned to detect an absolute difference in transfusion rate of 2% (5% in the standard care group, 3% in the cell salvage group, relative risk 0.6) with a power of 80% for a 2-sided test, and a type I error rate of 5% (for rate assumptions see the SALVO protocol).

All analyses were performed using Stata version 12 and on an intention-to-treat basis. For each primary and secondary outcome, we analysed all participants with non-missing data for that outcome. This approach is valid if data are missing at random (MAR) [27]. Our analysis plan specified that if more than 5% of primary outcome data were missing, we would conduct sensitivity analyses to investigate the impact of departures from the MAR assumption on our conclusions. Numbers of participants with missing outcome data are recorded in the results. Univariate and multivariable regression were used to estimate crude and adjusted odds ratios (ORs) for binary outcomes and mean differences for continuous outcomes, along with 95% confidence intervals (CIs). Adjusted risk differences for the primary outcome were calculated from multivariable logistic regression results using the ‘nlcom’ procedure in Stata. Number needed to treat (NNT) was calculated as 100 divided by the risk difference in percent. ‘Time to event’ variables were analysed using Cox proportional hazard regression to estimate the hazard ratio (HR). Multivariable models adjusted for stratification factors (with treatment centre as a random effect) and factors identified a priori to be prognostic for the primary outcome. The adjusted analysis was pre-specified as primary: such adjustment typically achieves substantial improvements in power, even when covariates are balanced [28].

We performed 2 pre-specified subgroup analyses: analyses of treatment effect by indication for cesarean section (elective versus emergency) and by treatment centre. The first of these was analysed by statistically testing for an interaction between indication for cesarean section and treatment. The second was analysed by testing for a random regression coefficient for the effect of treatment at different centres, in addition to a random intercept. Post hoc we conducted an analysis of treatment effect by normal versus abnormal placentation, also by testing for an interaction term.

We conducted 2 pre-specified sensitivity analyses: first, the primary analysis was redone excluding cases of placental abruption; second, we analysed the primary outcome where return of cell salvaged blood in the control group was reclassified as receiving a donor blood transfusion. Post hoc we also restricted the second sensitivity analysis so that only participants who received cell salvaged blood in the control group in an emergency setting were reclassified as having received donor blood.

A cost-effectiveness analysis was carried out from the perspective of the healthcare provider (UK National Health Service) [29] based on the principal clinical outcome of the trial, with the results expressed as cost per unit of donor blood transfusion avoided. A decision tree model was used that collated all the relevant resource use, cost, and outcome data collected prospectively during the trial to compare the overall cost-effectiveness of cell salvage with standard care. The resource use for both groups of the trial was estimated by prospectively evaluating the individual components of cell salvage and standard care (bottom-up costing). Unit cost data were then attached to the resource use. A probabilistic sensitivity analysis was carried out to explore the effects of the inherent uncertainty in parameter estimates on model results [30].

A trial steering committee and an independent data monitoring committee provided oversight to the study.

### Patient and public involvement

The UK National Childbirth Trust collaborated in the project by providing patient and public input through involvement in trial design and protocol development. Prior to this trial, a survey was conducted among women who received cell salvage, showing that they perceived the intervention as reassuring, safe, and preferable to donor blood transfusion (our primary outcome). A patient representative was a member of the trial steering committee to provide oversight and advice regarding recruitment, dissemination, and general trial management. We are planning to disseminate findings to participants in the form of a newsletter following primary publication of these results.

## Results

Between 4 June 2013 and 17 April 2016, 3,054 participants were recruited. The trial ended after inclusion and treatment of the originally planned sample of participants, with the discharge of the last patient on 21 April 2016.

**Fig 1:**
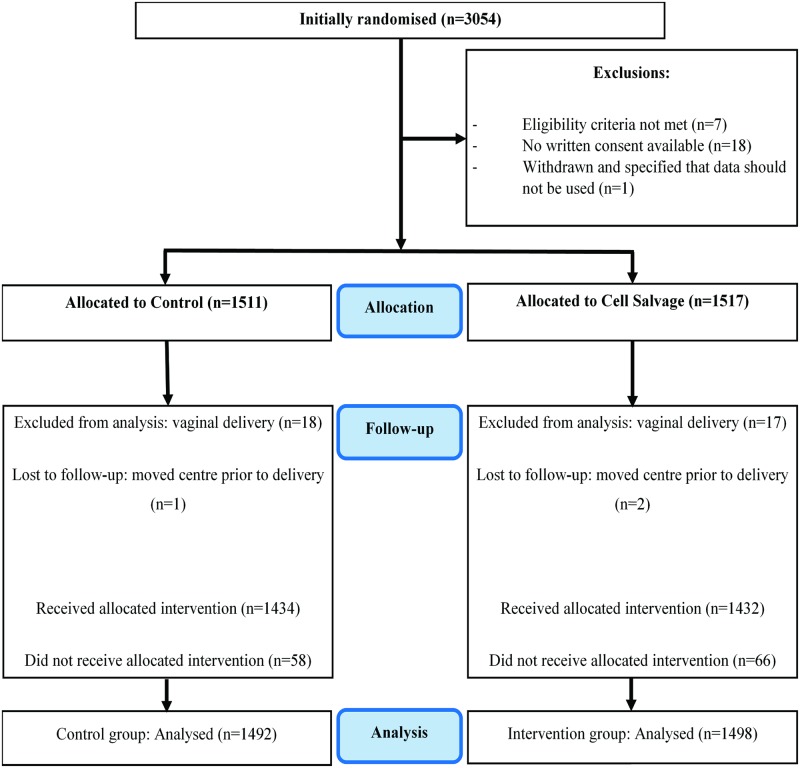
Participant enrolment and follow-up (CONSORT flow diagram).

After exclusions for eligibility and consent issues, 3,028 participants were randomly allocated to either control or intervention. Of these, 1,672 were scheduled for emergency and 1,356 for elective cesarean section. After excluding further participants due to vaginal delivery or transfer to another hospital, 1,492 participants remained in the control group and 1,498 in the intervention group for analysis (Fig 1). Baseline characteristics of participants were similar in the 2 groups (Table 1; additional characteristics are available in Table A in S1 Appendix).

**Table 1:** Characteristics of participants at baseline.

Characteristic	Control (*n* = 1,511)	Cell salvage (*n* = 1,517)
**Age at randomisation (years)**	31.8 (5.8)	31.6 (5.7)
**Preoperative haemoglobin (g/l)**	118.1 (11.5) [19]	118.4 (11.3) [11]
**Indication for cesarean**^1^		
Elective	687 (45.5%)	669 (44.1%)
Emergency	824 (54.5%)	848 (55.9%)
**Multiple birth**^1^		
Singleton	1,428 (94.5%)	1,428 (94.1%)
Twins or multiple	83 (5.5%)	89 (5.9%)
**Placentation**^1^		
Abnormal^2^	135 (8.9%)	136 (9.0%)
Normal	1,376 (91.1%)	1,381 (91.0%)
Placenta praevia	130 (8.6%)	133 (8.8%)
Abnormally invasive placenta	8 (0.5%)	4 (0.3%)
**Pre-eclampsia**	74 (4.9%)	69 (4.5%)
**Previous emergency cesarean**	602 (39.8%)	633 (41.7%)
**Previous elective cesarean**	241 (15.9%)	231 (15.2%)
**Placental abruption**	3 (0.2%)	2 (0.1%)
**Ethnicity**		
White	1,213 (80.3%)	1,219 (80.4%)
Mixed	23 (1.5%)	14 (0.9%)
Asian or Asian British	158 (10.5%)	173 (11.4%)
Black or Black British	67 (4.4%)	71 (4.7%)
Other	50 (3.3%)	40 (2.6%)
**Parity**		
0	571 (37.8%)	583 (38.4%)
1	556 (36.8%)	562 (37.0%)
2	240 (15.9%)	238 (15.7%)
3+	144 (9.5%)	134 (8.8%)

Data presented are *n* (%) or mean (SD) [*n* missing].

^1^Used as a stratification factor in randomisation (along with treatment centre).

^2^Placenta praevia and/or abnormally invasive placenta.

Adherence to the assigned intervention was 96.1% (1,434 participants) in the control group and 95.6% (1,432 participants) in the cell salvage group. In the cell salvage group, 50.8% had salvaged blood returned, averaging 259.9 ml (Table 2); there were 24 cases (1.6%) where the salvage machine was unavailable or out of order and 42 cases (2.8%) where the machine was simply not set up, in deviation from the protocol. In the control group, 15 participants (1.0%) had cell salvage used in an emergency and 43 participants (2.9%) had it set up from the start of the procedure, in deviation from the protocol.

**Table 2:** Summary of cell salvage use.

Item	Control (*n* = 1,492)	Cell salvage (*n* = 1,498)
***Cell salvage machine set-up***		
Machine set up	43 (2.9%)	1,432 (95.6%)
Emergency use	15 (1.0%)	0 (0.0%)
Machine not set up	1,434 (96.1%)	42 (2.8%)
Unavailable/out of order	0 (0.0%)	24 (1.6%)
Received allocated treatment	1,434 (96.1%)	1,432 (95.6%)
***If cell salvage set up (including emergency use) (n = 1*,*490)***		
**Number of suckers used**		
1	27 (48.2%)	829 (58.1%)
2	29 (51.8%)	598 (41.9%)
Missing	2	5
**Swabs washed**	21 (36.8%) [1]	781 (54.8%) [6]
**Size of centrifuge bowl used (ml)**^1^	183.2 (59.2) [2]	177.1 (59.8) [37]
**Leukocyte depletion filter used**	25 (43.9%) [1]	782 (54.9%) [7]
**Salvaged blood returned**	35 (60.3%) [0]	726 (50.8%) [3]
***If blood returned during cell salvage (n = 761)***		
Volume of blood returned to mother (ml)	288.4 (198.3)	259.9 (149.7)
***If no blood returned during cell salvage (n = 726)***		
**Reason for no return**		
No blood produced	14 (63.6%)	575 (88.9%)
Technical error	0 (0.0%)	25 (3.9%)
Other^2^	8 (36.4%)	47 (7.3%)
Missing	1	56

Data presented are *n* (%) or mean (SD) [*n* missing]. See Tables H and I in S1 Appendix for further summaries related to swab washing. Missing observations are not included in percentage calculations. Where variables are categorical, the number of participants with a missing value is listed in a separate row.

^1^Measure not applicable for sites with a continuous transfusion system only (control group: *n* = 22; cell salvage group: *n* = 180).

^2^Other reasons include ‘clinical decision’ (*n* = 7), ‘human error’ (*n* = 5), ‘meconium, infection risk, or contamination’ (*n* = 12), ‘minimal processed blood’ (*n* = 25), ‘patient declined’ (*n* = 2), ‘tubing trapped next to centrifuge bowl’ (*n* = 1), and ‘unclear’ (*n* = 3).

All participants had complete data on the primary outcome and on those characteristics specified as covariates in adjusted analyses. Overall, the transfusion rate was 3.5% in the control group versus 2.5% in the intervention group (adjusted OR 0.65, 95% CI 0.42 to 1.01, *p* = 0.056; adjusted risk difference −1.03, 95% CI −2.13 to 0.06; NNT 97, at the lower limit of 95% confidence NNT was 47 and at the upper limit the number needed to harm was 1,667) (Table 3). In the planned subgroup analysis, the transfusion rate was 4.6% in women assigned to control versus 3.0% in those assigned to cell salvage among emergency cesarean sections (adjusted OR 0.58, 95% CI 0.34 to 0.99), whereas it was 2.2% in women assigned to control versus 1.8% in women assigned to intervention among elective cesarean sections (adjusted OR 0.83, 95% CI 0.38 to 1.83) (interaction *p* = 0.46). The test for heterogeneity of treatment effect across treatment centres (random regression coefficient for centre) was non-significant (*p* = 0.09). In the exploratory subgroup analysis, the transfusion rate was 2.9% in women assigned to control versus 1.8% in those assigned to cell salvage among cesarean sections with normal placentation (adjusted OR 0.56, 95% CI 0.34 to 0.94), whereas it was 8.9% in women assigned to control versus 9.6% in women assigned to intervention among cesarean sections with abnormal placentation (adjusted OR 0.83, 95% CI 0.38 to 1.83) (interaction *p* = 0.28). The planned sensitivity analysis assuming that any return of cell salvaged blood in the control group was in place of a donor blood transfusion showed a reduction in the rate of participants requiring donor blood transfusion from 5.6% to 2.5% (adjusted OR 0.39, 95% CI 0.26 to 0.59, *p <* 0.001). A reduction was also observed when the sensitivity analysis was restricted to reclassifying only those who received salvaged blood in the control group for acute emergency blood loss (4.0% versus 2.5%, adjusted OR 0.56, 95% CI 0.36 to 0.86, *p* = 0.008).

**Table 3:** Effect of the intervention on donor blood transfusion (the primary outcome).

Analysis	Number (%)		Crude analysis			Adjusted analysis^1^		
	Control (*n* = 1,492)	Cell salvage (*n* = 1,498)	Risk difference percent (95% CI)	Intervention odds ratio (95% CI)	*p*-Value	Risk difference percent (95% CI)	Intervention odds ratio (95% CI)	*p*-Value
**Primary analysis**								
Received donor blood transfusion	52 (3.5%)	37 (2.5%)	−1.02 (−2.23, 0.20)	0.70 (0.46, 1.08)	0.10	−1.03 (−2.13, 0.06)	0.65 (0.42, 1.01)	0.056
**Sub-group analysis by indication for cesarean**								
Emergency cesarean (*n* = 1,641)	37 (4.6%)	25 (3.0%)					0.58 (0.34, 0.99)	
Elective cesarean (*n* = 1,349)	15 (2.2%)	12 (1.8%)					0.83 (0.38, 1.83)	
*p*-Value for interaction								0.46
**Sub-group analysis by placentation**^2^								
Normal placentation (*n* = 2,720)	40 (2.9%)	24 (1.8%)					0.56 (0.34, 0.94)	
Abnormal placentation (*n* = 270)	12 (8.9%)	13 (9.6%)					0.98 (0.42, 2.32)	
*p*-Value for interaction								0.28
**Sensitivity analysis**								
Excluding participants with placental abruption (cell salvage group: *n* = 2; control group: *n* = 3)	51 (3.4%)	37 (2.5%)	−0.95 (−2.17, 0.26)	0.72 (0.47, 1.10)	0.13	−0.97 (−2.07, 0.12)	0.67 (0.43, 1.03)	0.071
Assuming that return of cell salvaged blood in the control group avoided transfusions	83 (5.6%)	37 (2.5%)	−3.09 (−4.50, −1.69)	0.43 (0.29, 0.64)	<0.001	−2.50 (−3.86, −1.14)	0.39 (0.26, 0.59)	<0.001
Assuming that return of cell salvaged blood in the control group in an emergency avoided transfusions^2^	60 (4.0%)	37 (2.5%)	−1.55 (−2.82, −0.28)	0.60 (0.40, 0.92)	0.018	−1.53 (−2.72, −0.33)	0.56 (0.36, 0.86)	0.008

^1^Adjusted for stratification factors (elective versus emergency cesarean section, presence of abnormal placentation, singleton versus twins or multiple births, and treatment centre [as a random effect]) and other factors believed to be prognostic a priori (known placenta praevia and pre-eclampsia).

^2^Analysis was conducted post hoc.

All secondary outcomes had less than 5% missing data, except for fetomaternal haemorrhage (Table 4). There were small differences between groups for time to mobilisation (median 0.74 versus 0.72 days for the control and intervention group, respectively, adjusted HR 1.11, 95% CI 1.03 to 1.19, *p* = 0.006) and length of hospital stay (2.131 versus 2.126 days, adjusted HR 1.08, 95% CI 1.00 to 1.16, *p* = 0.050). For the subgroup of RhD-negative mothers with RhD-positive babies, women assigned to the intervention group had a greater rate of fetomaternal haemorrhage ≥2 ml than women assigned to the control group (10.5% [*n* = 9] versus 25.6% [*n* = 21], adjusted OR 5.63, 95% CI 1.43 to 22.14, *p* = 0.013). When blood was returned in this subgroup during cell salvage, 48% of participants (*n* = 15) experienced exposure to fetal blood, compared to 13% (*n* = 6) when blood was not returned (see Table D in S1 Appendix). There were no differences between groups in other secondary outcomes, including adverse events. Of 18 events related to cell salvage, 16 were associated with leukocyte depletion filter use. Two serious adverse reactions were reported: one patient experienced tachycardia and difficulty breathing following re-transfusion of cell salvaged blood, and another patient experienced sudden hypotension after transfusion of 600 ml of cell salvaged blood. Both events were classed by the local investigator as life-threatening and potentially related to the use of cell salvage, in particular to the use of a leukocyte depletion filter (which was not mandated by the study protocol). In both instances, patients recovered fully after cell salvage was discontinued. There was not a single case of amniotic fluid embolism in any instance of cell salvage use, with or without leukocyte depletion filters (details of adverse events are available in Tables E–G in S1 Appendix).

**Table 4:** Analysis of secondary outcomes.

Outcome	Measure	Control (*n* = 1,492)	Cell salvage (*n* = 1,498)	Crude analysis		Adjusted analysis^1^	
				Intervention OR, MD, or HR (95% CI)	*p*-Value	Intervention OR, MD, or HR (95% CI)	*p*-Value
**Secondary outcomes**							
Units of blood transfused^2^	Mean (SD)	2.65 (1.66)	2.70 (1.70)	MD 0.05 (−0.67, 0.76)	0.89	MD −0.12 (−0.80, 0.57)	0.74
Time to mobilisation (days)^3^^,^^4^	Median (IQR) [*n* missing]	0.74 (0.45) [49]	0.72 (0.45) [61]	HR 1.07 (0.99, 1.15)	0.079	HR 1.11 (1.03, 1.19)	0.006
Length of hospital stay (days)^3^^,^^5^	Median (IQR) [*n* missing]	2.13 (1.41) [24]	2.13 (1.37) [12]	HR 1.04 (0.97, 1.12)	0.26	HR 1.08 (1.00, 1.16)	0.050
**Safety outcomes**							
Postoperative haemoglobin level (g/l)^6^	Mean (SD) [*n* missing]	103.1 (12.1) [47]	103.8 (12.2) [61]	MD 0.74 (−0.15, 1.63)	0.10	MD 0.63 (−0.09, 1.35)	0.085
Fall in haemoglobin level (g/l)^6^	Mean (SD) [*n* missing]	15.0 (11.2) [65]	14.5 (11.1) [72]	MD −0.49 (−1.31, 0.33)	0.24	MD −0.68 (−1.40, 0.04)	0.066
Any adverse event experienced^7^	*n* (%) [*n* missing^8^]	191 (12.8%) [0]	199 (13.3%) [1]	OR 1.04 (0.84, 1.29)	0.69	OR 1.02 (0.81, 1.29)	0.84
Fetomaternal haemorrhage^9^	*n* (%) [*n* missing]	9 (10.5%) [33]	21 (25.6%) [51]	OR 2.95 (1.26, 6.89)	0.013	OR 5.63 (1.43, 22.14)	0.013
**MFI groups**^10^							
General fatigue	Mean (SD) [*n* missing]	12.7 (3.6) [52]	12.5 (3.6) [39]	MD −0.18 (−0.47, 0.12)	0.24	MD −0.18 (−0.47, 0.11)	0.22
Physical fatigue	Mean (SD) [*n* missing]	12.3 (3.9) [22]	12.3 (3.9) [31]	MD −0.05 (−0.36, 0.26)	0.75	MD −0.06 (−0.37, 0.25)	0.69
Reduced motivation	Mean (SD) [*n* missing]	9.6 (3.3) [36]	9.8 (3.4) [46]	MD 0.12 (−0.15, 0.40)	0.37	MD 0.13 (−0.14, 0.40)	0.36
Reduced activity	Mean (SD) [*n* missing]	11.3 (3.8) [42]	11.4 (3.6) [47]	MD 0.12 (−0.18, 0.43)	0.44	MD 0.12 (−0.18, 0.41)	0.45
Mental fatigue	Mean (SD) [*n* missing]	8.7 (3.6) [19]	8.4 (3.6) [41]	MD −0.28 (−0.57, 0.01)	0.061	MD −0.30 (−0.59, −0.01)	0.043

Analysis of transfusion reaction associated with allogeneic donor blood omitted due to observing only 1 event (control group).

^1^Adjusted for stratification factors (elective versus emergency cesarean section, presence of abnormal placentation, singleton versus twins or multiple births, and treatment centre [as a random effect]) and other factors believed to be prognostic a priori (known placenta praevia and pre-eclampsia).

^2^Analysis within the subgroup of participants who received donor blood.

^3^Taken from time of delivery.

^4^Test of proportional hazards assumption: crude analysis *p* = 0.67, adjusted analysis *p* = 0.18.

^5^Test of proportional hazards assumption: crude analysis *p* = 0.57, adjusted analysis *p* = 0.39.

^6^Adjusted analysis also adjusts for preoperative measurement, as well as the time the postoperative measurement was taken after delivery (log transformed), with mean imputation of missing values for both covariates. Please note that the decision to adjust for the latter was made by blinded members of the trial team after the signing off on the statistical analysis plan.

^7^See Tables E–G in S1 Appendix for further details on adverse events, adverse reactions, and serious adverse events.

^8^Missing observations are not included in percentage calculations.

^9^Measured by Kleihauer test and dichotomised into a result of <2 ml versus ≥2 ml. Analysis is within the subgroup of 270 RhD-negative participants with RhD-positive babies, of whom 252 had a Kleihauer test (119 in the control group, 133 in the cell salvage group). This measure was set to missing where results were not categorisable, e.g., where Kleihauer result was reported as <4 ml (control group: *n* = 25; cell salvage group: *n* = 42).

^10^Sum of MFI statement scores (where participants indicate level of agreement with a statement between 1 and 5) within fatigue categories. Higher scores indicate more fatigue.

HR, hazard ratio; MD, mean difference; MFI, Multidimensional Fatigue Inventory; OR, odds ratio.

The result of the cost-effectiveness analysis, based on the intention-to-treat analysis, showed an incremental cost-effectiveness ratio (ICER) of £8,110 (US$10,303; €9,711) per transfusion avoided for cell salvage compared to standard care. The probabilistic sensitivity analysis shows that although cell salvage was more effective than standard care for avoiding donor blood transfusion, it is uncertain whether it was less or more costly than standard care. Overall, if a decision-maker was willing to pay £50,000 (US$63,520; €59,869) to avoid a donor blood transfusion, the probability of cell salvage being cost-effective was 62% (see S1 Appendix for more detailed data).

## Discussion

This large, pragmatic, multicentre randomised trial showed that the routine use of cell salvage during cesarean section did not lead to a statistically significant reduction in the rate of donor blood transfusion in all women at risk of haemorrhage during cesarean section. Cell salvage was associated with increased maternal exposure to fetal blood among RhD-negative mothers. No other clinically relevant differences were observed in secondary outcomes. No cases of amniotic fluid embolism were observed, with or without leukocyte depletion filters. The cost-effectiveness of cell salvage is uncertain.

To our knowledge, our study is the largest randomised controlled trial in the area of cell salvage in obstetrics, and the only large-scale exploration of the clinical effectiveness and cost-effectiveness of cell salvage in cesarean section. It was prospectively registered, robustly conducted, independently monitored, rigorously analysed, and transparently reported. We recruited to target with independent data monitoring, had minimal patient or data loss, and achieved comparability at baseline. Compliance with assignment was generally excellent, but the deployment of cell salvage in the control group was a weakness as it could have potentially averted the use of donor transfusion, reducing the control event rate. We could not ethically prevent such action in emergencies, but we performed a sensitivity analysis reclassifying such cases as having experienced the primary outcome (donor blood transfusion), which showed an effect consistent in direction with the main result. Our audit to evaluate the risk of performance bias did not show imbalance in compliance with local transfusion policies. Our primary analysis followed the a priori statistical analysis plan, written before unblinding the randomised allocation, as agreed upon with our trial steering and data monitoring committees. It adjusted for the variables pre-specified. The usual rule of thumb for sample size in multivariable logistic regression of 10 cases per variable [31] was met in the adjusted analysis model. These methodological features should provide confidence in the validity and reliability of the findings. The diversity of our sample, in terms of cesarean indication, age, ethnicity, and geographic spread across many treatment centres, adds to generalisability. A *p*-value that is in the region of 0.05, regardless of the side of the significance threshold on which it lies, deserves careful consideration. It would be incorrect to conclude that the addition of further data would push the *p*-value below the threshold [32]. We believe our observations can justifiably be classed as modest [32], but not certain, evidence that can be useful in decision-making.

Our finding concerning the safety of cell salvage in cesarean sections shows that concerns about the risk of amniotic fluid embolism should not be a barrier to its deployment. The 2 serious adverse reactions observed are in keeping with known effects of leukocyte depletion filters [33]. If cell salvage is to be used, avoidance of these filters should be considered in order to reduce the risk of adverse reactions. Our finding concerning fetomaternal haemorrhage should be interpreted with caution. There were fewer than 10 events per variable in the model. This was in part because of large rates of missing data. Sensitivity analyses that assume worst- or best-case scenarios would inevitably give divergent results in this situation. It is debatable whether one could rely on accurately imputing missing outcomes from the data that were available. Despite these limitations, the risk of maternal exposure to fetal blood is a key issue for policies concerning obstetric use of cell salvage. There is a need to put mechanisms in place for maximising adherence to anti-D prophylaxis guidelines for the prevention of RhD red cell isoimmunisation. UK guidelines recommend a dose of 1,500 IU of anti-D following birth of an RhD-positive baby to an RhD-negative mother after cell salvage, with tests for fetomaternal haemorrhage to check if additional doses are needed [15].

The findings around the secondary outcome of fetomaternal haemorrhage highlight a need not only for long-term vigilance but also for research to determine the efficacy of anti-D prophylaxis, given that our study does not provide long-term follow-up data on RhD-negative mothers. The UK Serious Hazards of Transfusion haemovigilance scheme has flagged up the risk of sensitisation in women who do appear to have received appropriate prophylaxis [34]. Investigation is needed to determine if greater amounts of routine anti-D administration are required where cell salvage has been used in RhD-negative mothers. Additionally, the rate and severity of red cell isoimmunisation to rarer, non-RhD antibodies following cell salvage is unknown [16] and merits further study.

Concerning policy-making for deployment of cell salvage, its cost-effectiveness is going to be an issue for funders of services. Even if routine use of cell salvage was shown to be clinically effective, it is currently unlikely to be considered cost-effective for routine use in all indications for cesarean sections. Emergency cesarean sections have higher blood loss, and in these, cell salvage is not currently in routine use in practice. The potential for benefit in this group merits confirmation through additional research. The future benefit will depend on the extent to which cell salvage represents good value for money when changes occur in the rate of cesarean section, the rate of donor blood transfusion, the quality of the supply chain of donor blood for transfusion, and the contingency to address shocks on the supply of donor blood. Further delineation of cost-effectiveness in high-risk subgroups, particularly in settings with a limited supply of blood for transfusion, will be helpful in guiding decision-making.

## Supporting information

S1 Appendix Supplementary appendix. (PDF)

S1 Text SALVO trial protocol. (PDF)

S2 Text CONSORT checklist. (DOC)

## Abbreviations

CI: confidence interval
HR: hazard ratio
MFI: Multidimensional Fatigue Inventory
NNT: number needed to treat
OR: odds ratio
