# Supplementary material for: Cell salvage and donor blood transfusion during cesarean section: A pragmatic, multicentre randomised controlled trial (SALVO)
Source: PLoS Med. 2017 Dec 19;14(12):e1002471. doi: 10.1371/journal.pmed.1002471 (PMC5736174; doi:10.1371/journal.pmed.1002471)
Supplement: S1 Appendix — (PDF) [file pmed.1002471.s001.pdf]

SALVO Supplementary Appendix

Content:

1. *Economic Evaluation* 2

2. *Additional Figures* 5

3. *Additional Tables* 7

4. *References* 21

Figure A *Decision tree structure* 5

Figure B *Incremental cost-effectiveness scatterplot of cell salvage intended vs standard care intended for donor blood transfusion avoided* 6

Figure C *Cost-effectiveness acceptability curve for donor blood transfusion avoided* 6

Table A *Additional characteristics of participants at baseline* 7

Table B *Results concerning maternal RhD status* 8

Table C *Management of RhD-negative women with fetomaternal haemorrhage ≥ 2ml by Kleihauer test* 9

Table D *Fetomaternal haemorrhage by sucker use and by return of salvaged blood* 10

Table E *Detail of adverse events* 11

Table F *Further detail for events potentially related to cell salvage* 15

Table G *Further detail for serious adverse events* 16

Table H *Summaries specific to swab washing* 18

Table I *Analysis of primary outcome: Analysis by swab washing* 18

Table J *Intraoperative resource use and costs* 19

Table K *Postoperative resource use and costs* 20

Table L *Results for the base-case analysis* 20

1. *Economic Evaluation*

Methods

To compare the costs and outcomes of intraoperative cell salvage and standard care in the SALVO trial, a decision analytic model was deemed the most suitable method of presenting the alternative pathways and collating the data for analysis and sensitivity analysis. The economic evaluation took the form of a cost-effectiveness analysis from the perspective of the healthcare provider based on the principal clinical outcome of the trial. The main comparison is the use of cell salvage versus standard care. The results are reported in terms of the additional cost per donor blood transfusion avoided by using cell salvage compared to standard care. Standard care is defined in the trial literature as "transfusion of donor blood according to standard local guidelines". Given the objective of the trial and the duration of follow up, only a within trial economic analysis was carried out and outcomes beyond this point were not considered relevant.

<u>Model Structure</u>
A decision tree model was developed in TreeAge Pro 2016 (TreeAge Software, Inc., Williamstown, MA, USA). The model pathways represent that of the trial in which patients undergoing a caesarean section were randomised to receive either cell salvage or standard care. Figure A shows the model starts with the choice of transfusion strategies considered in the SALVO trial:

- Cell salvage
- Standard care

Women allocated to either transfusion strategy have a possibility of receiving the treatment to which they were allocated or not. In both pathways, if the cell salvage machine was switched on, women have a possibility of receiving cell salvage, either on its own or in combination with donor blood transfusion. There is also a possibility that the woman may not require a transfusion.

The pathways of the model represent, as far as possible, the clinical procedures carried out in the study. The model combines the probability of a woman following a particular path and the associated costs. Probabilities were obtained from the trial and attached to each pathway. The cost and outcome measures that were incorporated into the model were collected prospectively during the SALVO trial using forms filled out at the pre-, intra-, and post-operative phase and at the time of discharge from hospital. Intra-operative resource use and costs were estimated as the mean cost per caesarean section procedure for each treatment pathway in the model and postoperative resource use and costs were estimated as the mean cost per patient in both treatment strategies represented in the model.

<u>Data</u>

Resource use and costs
The resource use for both arms of the trial was estimated by evaluating the individual components of these procedures (bottom-up costing). Unit cost data was then attached to the resource use. Data was collected on all major NHS resource use for each patient using the trial case report forms. Costs are reported in 2014-15 British Pounds (£).

For the analysis, intraoperative and postoperative resource use data were obtained from the SALVO trial. The main resource use monitored included: equipment and disposables required for the cell salvage procedure; additional staff called into theatre solely for the purposes of cell salvage; drugs used in the caesarean section procedure; the use of donor blood transfusion to manage haemorrhage and its consequences; the use of salvaged blood transfusion to manage haemorrhage and its consequences; length and type of hospital inpatient stay including additional treatment required attributed to the caesarean section procedure.

Intraoperative costs were estimated for each item to arrive at a mean cost per caesarean procedure for each treatment pathway in the model. To estimate the cost of a caesarean procedure some costs were calculated at the patient level, e.g. swab washing, and some at the procedural level, e.g. drugs used in the caesarean section procedure (see Table J). Postoperative costs were estimated for each item based on their occurrence in each branch of the model to arrive at a mean cost per patient for each branch (see Table K).

Outcomes

The outcome of interest in the trial was the use of donor blood transfusion in response to haemorrhage and its consequences.

Assumptions

It was necessary to make the following pragmatic assumptions before the analysis could be carried out:

(i) All of the centres involved in the trial were assumed to have the same expertise and to have followed similar protocols in the management of patients.

(ii) It was assumed that all centres performing cell salvage used consumables and that one collection set and one processing pack were used per cell salvage procedure. Costs for equipment and disposables were obtained for a Haemonetics Cell Saver 5 machine. Where swab washing occurred, it was assumed that the swabs were washed in one litre of saline.[1]

(iii) Where the cell salvage machine was switched on, it was assumed that running costs would be incurred and a collection set would be used even if no salvaged blood was returned to the patient. It was also assumed that heparin and saline would be used prior to collection.[2]

(iv) We based our analysis on the staff type most frequently called into theatre in the trial and assumed the lowest possible cost within this job band distinction.

(v) The threshold setting on a cell salvage machine can be set to engage for blood above a certain volume, and in this study, trial centres displayed variance in the minimum volume threshold they selected. Guidance in this trial states that all salvaged blood produced by the machines should be returned to the patient. Therefore, this analysis assumed that all minimum threshold settings were disengaged. The collection of all shed blood was considered, regardless of whether that blood was subsequently returned to the patient.

(vi) All units transfused were assumed to be red blood cells (RBC).[2] The mean number of units transfused per patient was rounded up to account for the fact that any remaining blood in a bag would be disposed of.

(vii) Where patients received level 0 care when admitted to Higher Level of Care (HLC), it was assumed that their needs could be met through general ward care. It was assumed that level 1 care was 25% more expensive per day than level 0 care and level 2 care was 25% more expensive than level 1 care.

(viii) It was assumed that non serious adverse events deemed relevant to the procedure would have limited or zero resource impact. It was assumed that in the case of an acute transfusion reaction, the transfusion would be discontinued.[3]

(ix) In this study, the health of the infant was not considered relevant to the intervention. Information relating to the clinical status and care of the infant was therefore not included in the analysis.

Analysis

Given the objectives of the trial and the duration of follow-up, a within trial economic analysis was carried out. The analysis took the perspective of the NHS following the current recommendation from NICE.[4] The main economic analysis was a cost-effectiveness analysis with results expressed as cost per donor transfusion avoided.

In the analysis the base-case was based on the intention-to-treat (ITT) principle. In this method, patients are compared within the treatment groups to which they were originally randomised irrespective of the treatment received.[5] This method of analysis allows the estimates to follow real-life scenarios in which patients may not always receive the planned treatment. Not using ITT analysis can often exaggerate the benefits of a given intervention.[5]

Sensitivity analysis

Probabilistic sensitivity analyses (PSAs) were carried out for the analysis to explore the effects of the inherent uncertainty in parameter estimates on model results. Monte Carlo simulation was used to sample from these distributions to allow the effect of parameter uncertainty to be evaluated. This involved 1000 repeated random draws from the distributions to indicate how variation in the model parameters would affect the results and hence illustrate the decision uncertainty. Beta distributions were used for probability data and Gamma distributions for costs.[6,7]

The results of the analyses are presented in terms of an incremental cost-effectiveness ratio (ICERs), which reflects the additional cost per donor blood transfusion avoided of cell salvage compared with standard care. The results

of the PSA are presented using a scatterplot and cost-effectiveness acceptability curve (CEAC) to reflect sampling variation and uncertainties in the appropriate threshold cost-effectiveness value.

Results

The results of the analysis are shown in Table L. The strategy in which standard care was intended was the least costly, with the average cost per patient estimated at £1,244. However, the cell salvage intended arm was only slightly more expensive with the average cost per patient estimated at £1,327. The cell salvage intended strategy was the most effective at avoiding a transfusion. The estimated ICER for the cell salvage intended strategy compared with standard care was £8,110 per donor blood transfusion avoided. This means that it would cost an additional £8,110 to avoid a donor blood transfusion through cell salvage compared to standard care.

The scatterplot (Figure B) shows the modelled uncertainty in the cost and effectiveness of the cell salvage intended strategy compared with the standard care intended strategy from 1,000 Monte Carlo simulations. In this, the ICER of each simulation is plotted on the cost-effectiveness plane providing information about the joint density of the differences in cost and effectiveness between the two strategies. From Figure B, it is evident that although cell salvage is a more effective transfusion strategy, it is uncertain whether it is less or more costly than standard care. The CEAC (Figure C) shows that the probability that cell salvage is cost-effective increases as the willingness to pay for a donor blood transfusion avoided increases. If a decision maker was willing to pay £50,000 to avoid a donor blood transfusion, the probability of cell salvage being cost-effective is 62%.

2. Additional Figures

Figure A Decision tree structure

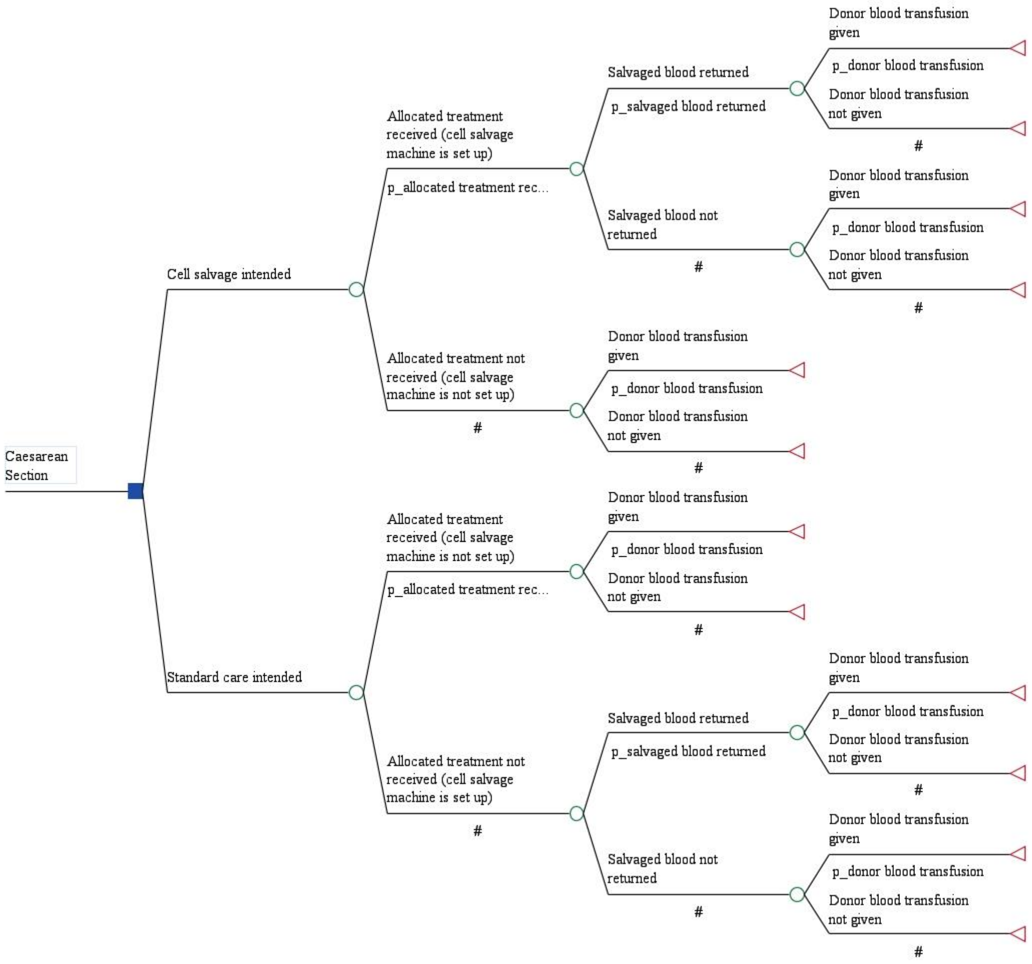

Figure B Incremental cost-effectiveness scatterplot of cell salvage intended vs standard care intended for donor blood transfusion avoided

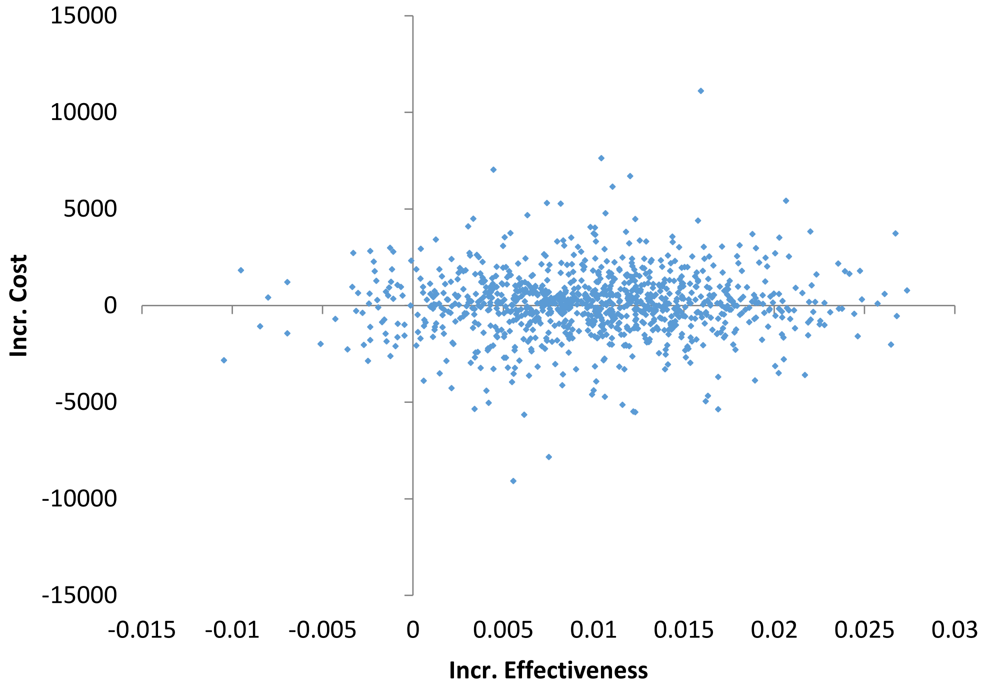

Figure C Cost-effectiveness acceptability curve for donor blood transfusion avoided

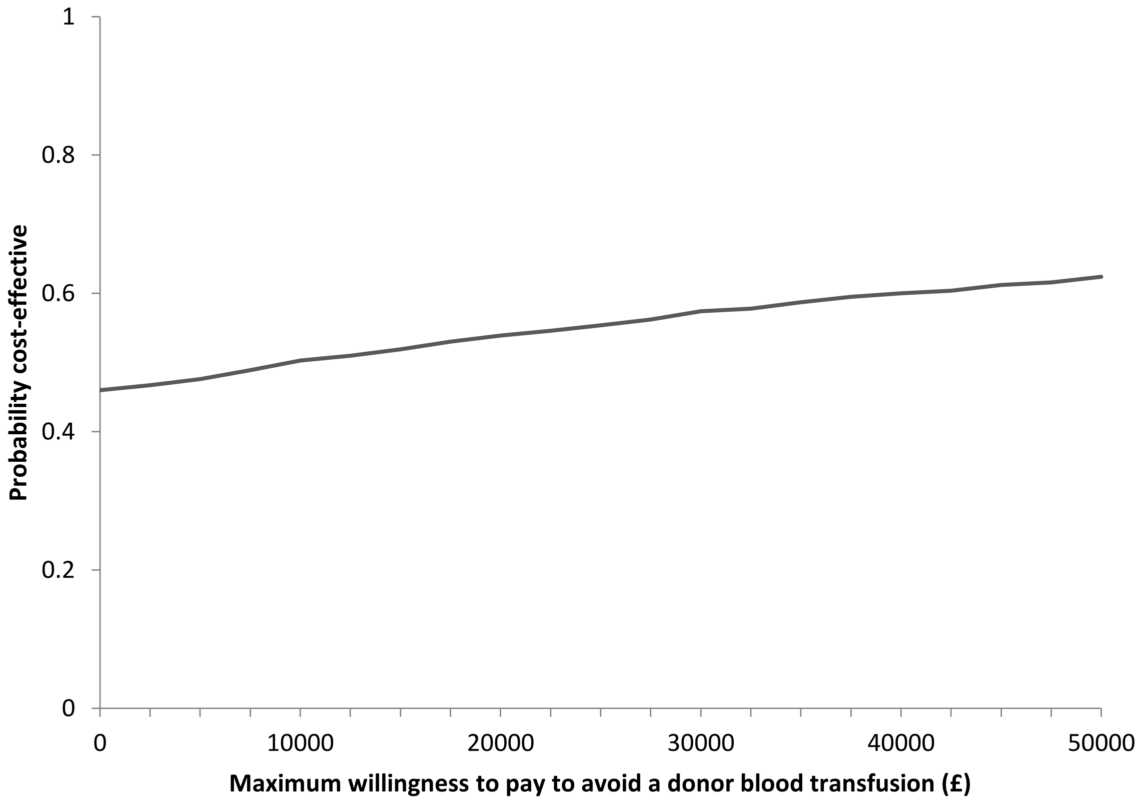

3. *Additional Tables*

Table A Additional characteristics of participants at baseline

	Control (n=1511)	Cell Salvage (n=1517)
**Centre**		
Birmingham Heartlands Hospital	41 (2.7%)	44 (2.9%)
Birmingham Women's Hospital	7 (0.5%)	6 (0.4%)
Croydon University Hospital	48 (3.2%)	48 (3.2%)
Derriford Hospital Plymouth	57 (3.8%)	59 (3.9%)
Hinchingbrooke Hospital	83 (5.5%)	84 (5.5%)
James Cook University Hospital	109 (7.2%)	108 (7.1%)
Leicester General Hospital	5 (0.3%)	4 (0.3%)
Leicester Royal Infirmary	78 (5.2%)	75 (4.9%)
Norfolk and Norwich University Hospital	5 (0.3%)	5 (0.3%)
Northwick Park Hospital	13 (0.9%)	15 (1.0%)
Nottingham City Hospital	15 (1.0%)	16 (1.0%)
Queens Hospital Romford	60 (4.0%)	58 (3.8%)
Queens Medical Centre Nottingham	15 (1.0%)	13 (0.9%)
Royal Hallamshire Hospital Sheffield	138 (9.1%)	139 (9.2%)
Royal London Hospital	84 (5.6%)	87 (5.7%)
Royal Stoke University Hospital, Stoke-on-Trent	72 (4.8%)	73 (4.8%)
Royal United Hospital Bath	88 (5.8%)	87 (5.7%)
Royal Victoria Infirmary Newcastle	119 (7.9%)	116 (7.7%)
Simpson Centre Edinburgh	47 (3.1%)	51 (3.4%)
Singleton Hospital Swansea	84 (5.6%)	88 (5.8%)
St. Michaels Hospital Bristol	26 (1.7%)	21 (1.4%)
Sunderland Royal Hospital	192 (12.7%)	190 (12.5%)
Torbay Hospital	28 (1.9%)	30 (2.0%)
West Middlesex University Hospital	49 (3.2%)	52 (3.4%)
Whipps Cross University Hospital	29 (1.9%)	27 (1.8%)
Whiston Hospital	19 (1.3%)	21 (1.4%)
**Gravidity**		
1	420 (27.8%)	441 (29.1%)
2	467 (30.9%)	465 (30.6%)
3+	624 (41.3%)	611 (40.3%)

Data presented are n (%)

Table B **Results concerning maternal RhD status**

		Control (n=1492)	Cell Salvage (n=1498)
**RhD-Negative Mother With RhD-Positive Baby**	n (%)	130 (8.7%)	140 (9.3%)
**If Mother Negative and Baby Positive (n=270)**			
**Anti-D Prophylaxis Administered?**	n (%)	129 (99.2%)	138 (98.6%)
**Anti-D Prophylaxis Dose (IU)**			
500	n (%)	59 (46.1%)	78 (56.5%)
1500	n (%)	67 (52.3%)	56 (40.6%)
Other(1)	n (%)	2 (1.6%)	4 (2.9%)
Missing(2)	n	1	0
**Kleihauer Test Performed?**	n (%) [n missing]	119 (92.2%) [1]	133 (95.0%) [0]
**Fetomaternal haemorrhage (Kleihauer test ≥2ml)**	n (%)	9 (10.5%)	21 (25.6%)
**Sample Sent For Flow Cytometry(3)**	n (%)	1 (33.3%)	9 (75.0%)
**Repeat Kleihauer Test Performed?(4)**	n (%)	1 (50.0%)	6 (100.0%)
**Further Anti-D Prophylaxis Administered?(4)**	n (%) [n missing]	1 (100.0%) [1]	1 (16.7%) [0]
**Further Anti-D Prophylaxis Dose (IU)**			
250	n (%)	0 (0.0%)	1 (100.0%)
1500	n (%)	1 (100.0%)	0 (0.0%)

(1) Other doses include Control group: 1250, 4000; Cell Salvage group: 1000, 1000, 4500, 5000. See Table C for details.
(2) Missing observations are not included in percentage calculations. Where variables are categorical, missing values are listed in a separate row, but are similarly not included in percentage calculations.
(3) Measure only collected for participants with Kleihauer >2ml (Control group: n=3, Cell Salvage group n=12)
(4) Measure only collected for participants with Kleihauer >4ml (Control group: n=2, Cell Salvage group: n=6). See Table C for details.

Table C Management of RhD-negative women with fetomaternal haemorrhage ≥ 2ml by Kleihauer test

FMH by Kleihauer (ml)	Anti-D dose (IU)	Flow cytometry undertaken[(1)]	Flow cytometry result (ml)	Repeat Kleihauer undertaken[(2)]	Blood returned during cell salvage?
**Cell salvage group (n=21)**					
26	4500	Yes	26	Yes	Yes
11	1500	Yes	9	Yes	Yes
10	1500	Yes	10	Yes	Yes
6	1000	Yes	5	Yes	No
6	1000	Yes	6	Yes	Yes
5	1500	Yes	12	Yes[(3)]	Yes
4	5000	No	-		No
4	500	Yes	2		No
3	500	No	-		Yes
3	None	No	-		No
>2	500	Yes	2		Yes
>2	1500	Yes	7		Yes
2	1500				Yes
2	1500				No
2	1500				Yes
2	500				Yes
2	500				Yes
2	500				Yes
2	500				Yes
2	500				Yes
2	500				No
**Control group (n=9)**					
37	4000	Yes	37	Yes[(3)]	Not set up
>4	1500	No	-	No	Not set up
3	500	No	-		Not set up
2	500				Not set up
5pts with 2	1500				Not set up

FMH: Fetomaternal haemorrhage.

(1) Flow cytometry data was only collected for Kleihauer > 2ml.

(2) Repeat Kleihauer data was only collected for Kleihauer > 4ml.

(3) Repeat anti-D also administered.

Table D **Fetomaternal haemorrhage by sucker use and by return of salvaged blood**

	**One Sucker Used (n=53)**	**Two Suckers Used (n=24)**
**Fetomaternal Haemorrhage (1)**	15 (28.3%)	6 (25.0%)
	**No Blood Returned (n=46)**	**Blood Returned (n=31)**
**Fetomaternal Haemorrhage (2)**	6 (13.0%)	15 (48.4%)

Data presented are n (%)

(1) Measured by Kleihauer test and dichotomised into a result of <2ml vs. ≥2ml. Summaries within participants who had the cell salvage machine set up (including Emergency use), for those with complete data on fetomaternal haemorrhage and sucker use

(2) Summaries within participants who had the cell salvage machine set up (including Emergency use), for those with complete data on fetomaternal haemorrhage and return of blood during cell salvage

Table E **Detail of adverse events**

	Control (n=1492)	Cell Salvage (n=1498)
**Any Adverse Event Experienced**	191 (12.8%) [0]	199 (13.3%) [1]
**Total Adverse Events**	220	233
**Breakdowns Per Adverse Event (n=453)**		
**Adverse Event Severity**		
Mild	89 (40.5%)	101 (43.3%)
Moderate	88 (40.0%)	92 (39.5%)
Severe	34 (15.4%)	35 (15.0%)
Life-threatening	9 (4.1%)	4 (1.7%)
Fatal	0 (0.0%)	1 (0.4%)
**Adverse Event Relatedness To Intervention (If Cell Salvage Set Up (Including Emergency Use) (n=238))**		
Unrelated	8 (57.1%)	160 (71.4%)
Unlikely	5 (35.7%)	47 (21.0%)
Possible(2)	1 (7.1%)	14 (6.3%)
Probable(2)	0 (0.0%)	2 (0.9%)
Definite(2)	0 (0.0%)	1 (0.4%)
**Is the Adverse Event Serious(3)**	20 (9.1%)	15 (6.4%)
**Adverse Event Descriptions(4) by System Organ Class**		
**Blood and lymphatic system disorders**		

	Control (n=1492)	Cell Salvage (n=1498)
Thrombocytopaenia	0 (0.0%)	3 (1.3%)
Anaemia	2 (0.9%)	6 (2.6%)
**Cardiac disorders**		
Sinus tachycardia	0 (0.0%)	5 (2.2%)
Hypotension	2 (0.9%)	2 (0.9%)
Supraventricular tachycardia	1 (0.5%)	1 (0.4%)
**Gastrointestinal disorders**		
Diarrhoea	0 (0.0%)	1 (0.4%)
Ileus	4 (1.8%)	3 (1.3%)
Incontinence	0 (0.0%)	1 (0.4%)
**General disorders and administration site conditions**		
Pain	4 (1.8%)	3 (1.3%)
Non-cardiac chest pain	1 (0.5%)	0 (0.0%)
**Immune system disorders**		
Reaction to cell salvaged blood	0 (0.0%)	5 (2.2%)
Reaction to donor blood	1 (0.5%)	0 (0.0%)
Allergic reaction	1 (0.5%)	0 (0.0%)
**Infections and infestations**		
Lung infection	2 (0.9%)	0 (0.0%)
Wound infection	5 (2.3%)	6 (2.6%)
Uterine infection	1 (0.5%)	2 (0.9%)
Sepsis	11 (5.0%)	11 (4.7%)
Unknown source	12 (5.5%)	21 (9.0%)
**Injury, poisoning and procedural complications**		
Wound dehiscence	1 (0.5%)	2 (0.9%)
**Metabolism and nutrition disorders**		
Hyperglycaemia	0 (0.0%)	1 (0.4%)
**Musculoskeletal and connective tissue disorders**		

	Control (n=1492)	Cell Salvage (n=1498)
Pain in extremity	0 (0.0%)	2 (0.9%)
Back pain	1 (0.5%)	0 (0.0%)
**Nervous system disorders**		
Presyncope	2 (0.9%)	3 (1.3%)
Seizure	2 (0.9%)	1 (0.4%)
Limb weakness	0 (0.0%)	1 (0.4%)
**Pregnancy, puerperium and perinatal conditions - Other**		
Hypertensive disease of pregnancy	32 (14.6%)	34 (14.6%)
Uterine atony	1 (0.5%)	0 (0.0%)
Placental abnormality	2 (0.9%)	1 (0.4%)
Fetomaternal haemorrhage	1 (0.5%)	0 (0.0%)
**Renal and urinary disorders**		
Urinary retention	3 (1.4%)	2 (0.9%)
Oliguria	3 (1.4%)	0 (0.0%)
Chronic kidney disease	0 (0.0%)	1 (0.4%)
Prolonged catheterisation	1 (0.5%)	3 (1.3%)
Proteinuria	1 (0.5%)	1 (0.4%)
Hematuria	1 (0.5%)	1 (0.4%)
**Reproductive system and breast disorders**		
Fibroids	1 (0.5%)	0 (0.0%)
Uterine haemorrhage	106 (48.4%)	93 (39.9%)
**Respiratory, thoracic and mediastinal disorders**		
Cough	0 (0.0%)	1 (0.4%)
Dyspnea	1 (0.5%)	0 (0.0%)
Hypoxia	1 (0.5%)	1 (0.4%)
Pulmonary edema	0 (0.0%)	1 (0.4%)
Sleep apnea	1 (0.5%)	0 (0.0%)
**Skin and subcutaneous tissue disorders**		

	Control (n=1492)	Cell Salvage (n=1498)
Pruritus	0 (0.0%)	1 (0.4%)
**Surgical and medical procedures - Other**		
Anaesthetic complication	3 (1.4%)	1 (0.4%)
Surgical complication	5 (2.3%)	2 (0.9%)
Wound haematoma	1 (0.5%)	5 (2.2%)
**Vascular disorders**		
Venous eczema	0 (0.0%)	1 (0.4%)
Hypertension	1 (0.5%)	3 (1.3%)
Thromboembolic event	1 (0.5%)	1 (0.4%)
**Missing(1)**	1	0

Data presented are n (%) [n missing]

(1) Missing observations are not included in percentage calculations. Where variables are categorical, missing values are listed in a separate row, but are similarly not included in percentage calculations

(2) For further detail see Table F

(3) For further detail see Table G

(4) Descriptions are coded by the trial team

Table F **Further detail for events potentially related to cell salvage**

Adverse Event Relatedness to Intervention	Allocation	System Organ Class of Adverse Event	Adverse Event Description
Possible	Control	Reproductive system and breast disorders	Uterine haemorrhage
Possible	Cell Salvage	Cardiac disorders	Hypotension
Possible	Cell Salvage	Immune system disorders	Reaction to cell salvaged blood
Possible	Cell Salvage	Immune system disorders	Reaction to cell salvaged blood
Possible	Cell Salvage	Infections and infestations	Sepsis
Possible	Cell Salvage	Infections and infestations	Unknown source
Possible	Cell Salvage	Infections and infestations	Unknown source
Possible	Cell Salvage	Infections and infestations	Unknown source
Possible	Cell Salvage	Infections and infestations	Unknown source
Possible	Cell Salvage	Infections and infestations	Unknown source
Possible	Cell Salvage	Infections and infestations	Unknown source
Possible	Cell Salvage	Reproductive system and breast disorders	Uterine haemorrhage
Possible	Cell Salvage	Reproductive system and breast disorders	Uterine haemorrhage
Possible	Cell Salvage	Surgical and medical procedures - Other	Wound haematoma
Possible	Cell Salvage	Infections and infestations	Wound infection
Probable	Cell Salvage	Immune system disorders	Reaction to cell salvaged blood
Probable	Cell Salvage	Immune system disorders	Reaction to cell salvaged blood
Definite	Cell Salvage	Immune system disorders	Reaction to cell salvaged blood

Table G Further detail for serious adverse events

Description[1] of Serious Adverse Event	Allocation	Reason for Seriousness	Serious Adverse Event Relatedness to Intervention
Bladder damage during surgery[2]	Control	Hospitalisation > 7 days	Unrelated
Concealed obstetric haemorrhage	Control	Life-threatening	Unrelated
HELLP Syndrome	Control	Other	Unrelated
Infection of unknown origin	Control	Hospitalisation > 7 days	Unrelated
Massive obstetric haemorrhage	Control	Life-threatening	Unrelated
Massive obstetric haemorrhage	Control	Life-threatening	Unlikely
Massive obstetric haemorrhage	Control	Life-threatening	Unrelated
Massive obstetric haemorrhage	Control	Life-threatening	Unrelated
Massive obstetric haemorrhage	Control	Life-threatening	Unrelated
Massive obstetric haemorrhage	Control	Life-threatening	Unrelated
Massive obstetric haemorrhage	Control	Life-threatening	Unrelated
Pneumonia	Control	Hospitalisation > 7 days	Unrelated
Pre-eclampsia	Control	Hospitalisation > 7 days	Unrelated
Pre-eclampsia	Control	Hospitalisation > 7 days	Unrelated
Pulmonary embolism and obstetric haemorrhage	Control	Life-threatening	Unrelated
Sepsis	Control	Hospitalisation > 7 days	Unrelated
Vertebral disc prolapse	Control	Disability/incapacity	Unlikely
Wound complication	Control	Hospitalisation > 7 days	Unrelated
Bowel obstruction, caecal gangrene	Cell Salvage	Hospitalisation > 7 days	Unrelated
Bowel perforation, sepsis, multi-organ failure	Cell Salvage	Fatal	Unrelated
Fetal congenital abnormality[3]	Cell Salvage	Congenital abnormality / birth defect	Unrelated
Fetal congenital abnormality[3]	Cell Salvage	Congenital abnormality / birth defect	Unrelated
Fetal epidermolysis bullosa[3]	Cell Salvage	Congenital abnormality / birth defect	Unrelated
Hypertension	Cell Salvage	Hospitalisation > 7 days	Unrelated
Massive obstetric haemorrhage	Cell Salvage	Life-threatening	Unlikely
Massive obstetric haemorrhage	Cell Salvage	Life-threatening	Unrelated
Palpitations and shortness of breath. Postpartum echocardiogram suggested mild left ventricular systolic dysfunction	Cell Salvage	Hospitalisation > 7 days	Unlikely
Pre-eclampsia	Cell Salvage	Hospitalisation > 7 days	Unrelated
Pre-eclampsia	Cell Salvage	Hospitalisation > 7 days	Unrelated
Pre-existing atrial fibrillation and wound complication	Cell Salvage	Hospitalisation > 7 days	Unrelated

Description[1] of Serious Adverse Event	Allocation	Reason for Seriousness	Serious Adverse Event Relatedness to Intervention
Reaction to salvaged blood or leukocyte depletion filter (hypotension)	Cell Salvage	Life-threatening	Probably
Reaction to salvaged blood or leukocyte depletion filter (tachycardia, dyspnoea)	Cell Salvage	Life-threatening	Definitely
Sepsis	Cell Salvage	Hospitalisation > 7 days	Unlikely
Sepsis	Cell Salvage	Hospitalisation > 7 days	Unlikely
Stillbirth[3]	Cell Salvage	Congenital abnormality / birth defect	Unrelated
Wound complication	Cell Salvage	Hospitalisation > 7 days	Unrelated
Wound complication	Cell Salvage	Hospitalisation > 7 days	Unrelated

(1) Descriptions are coded by the trial team.

(2) Participant had 3 adverse events which were ticked as serious, all falling under the serious adverse event described.

(3) Serious adverse events not included in Table E as they concern the baby, not the mother.

Table H **Summaries specific to swab washing**

		Swabs Not Washed (n=681)	Swabs Washed (n=802)
**Salvaged Blood Returned**	n (%) [n missing(1)]	109 (16.0%) [1]	651 (81.3%) [1]
**Volume of Blood Returned to Mother (ml)**	mean (sd)	32.8 (100.5)	219.3 (169.8)

sd: Standard Deviation

(1) Missing observations are not included in percentage calculations

Table I **Analysis of primary outcome: Analysis by swab washing**

		Swabs Not Washed (n=681)	Swabs Washed (n=802)	Crude Odds Ratio (95% CI)	P-Value - Crude Analysis	Adjusted(1) Odds Ratio (95% CI)	P-Value - Adjusted Analysis
**Donor Blood Transfusion(2)**	n (%)	18 (2.6%)	18 (2.2%)	0.85 (0.44, 1.64)	0.62	0.79 (0.39, 1.57)	0.50

CI: Confidence Interval

(1) Adjusted for stratification factors (elective vs. emergency caesarean section, presence of abnormal placentation, singleton vs. twins or multiple births, recruitment centre (as a random effect)) and other factors believed to be prognostic a-priori (known placenta praevia, pre-eclampsia).

(2) Analysis within participants who had the cell salvage machine set up (including Emergency use), for those with complete swab washing data.

Table J Intraoperative resource use and costs

Item	Resource Use		Unit Cost	Mean cost per procedure		Assumption / Working	Source
	Cell salvage (n=1498)	Control (n=1492)		Cell salvage (n-=1498)	Control (n=1492)		
Running costs	1432	58	£6.14	£6.14	£6.14	Based on annual maintenance costs for Haemonetics Cell Saver 5 machine and estimated annual usage	UHB, personal communication (Aug 2016) NICE costing statement blood transfusion (Nov 2015) [2]
Collection Set	1	1	£41.71	£41.71	£41.71	Based on the assumption that one collection set is used per procedure	NHS Supply Chain Catalogue (accessed Aug 2016): Autotransfusion resevoir 3 litre [8]
Processing Pack	1	1	£77.00	£77.00	£77.00	Based on the assumption that one processing pack is used per procedure	NHS Supply Chain Catalogue (accessed August 2016): Intraoperative autologous blood system cell saver 5+ bowl set 125ml [8]
Leukocyte depletion filter	782	25	n/a	n/a	n/a	Cost not included in the analysis as leukocyte depletion filter included in the collection set for Haemonetics Cell Saver 5 machine	NHS Supply Chain Catalogue (accessed August 2016): Autotransfusion resevoir 3 litre [8]
Additional sucker	598	29	£15.41	£6.43	£7.70	Mean cost based on the number of additional suckers used in each treatment arm / total number of patients who received cell salvage	NHS Supply Chain Catalogue (accessed August 2016): Aspiration & anticoagulation line Cell Saver. £308.02 for 20 [8]
Swab washing	781	21	£0.80	£0.44	£0.29	Mean cost based on the number of times swabs were washed in each treatment arm / total number of patients who received cell salvage	ICS Factsheet 1 Swab Washing March 2015,[1] based on the cost of 1L of sodium chloride 0.9%, BNF [9]
Staff	83.65 (min)	88.09 (min)	£0.72 (min)	£11.57	£12.03	Based on the staff type most frequently called into theatre.	Unit cost for hospital based nurse, band 5, PSSRU unit costs 2015 (costs include qualifications) [10]
Saline (litres)	2	2	£0.80	£1.60	£1.60	Based on the assumption that 2 litres of saline would be administered to all patients undergoing cell salvage prior to collection[2]	Based on the cost of 1L of sodium chloride 0.9%, BNF [9]
Heparin sodium (30,000 IU)	2	2	£10.60	£21.20	£21.20	Based on the assumption that 60,000 iu heparin would be administered to all patients undergoing cell salvage prior to collection[2]	Based on the cost of 1ml amp of heparin sodium 25,000 iu/ml and 1ml amp of heparin sodium 5,000 iu/ml, BNF [9]
Anti-D (500 IU)	1	1	£33.75	£3.04	£3.04	Based on the assumption that all D negative women delivering a D positive baby receive at least 500 IU of anti-D. [11] Mean cost per procedure based on the probability of a woman requiring anti-D in each treatment arm (0.09)	Based on the cost of 500-unit vial of anti-D immunoglobulin, BNF [9]
Anti-D (1500 IU)	1	1	£58.00	£5.22	£5.22	Based on the assumption that women who receive cell salvage are offered 1500 IU of anti-D [11]. Mean cost per procedure based on the probability of a woman requiring anti-D in each treatment arm (0.09)	Based on the cost of 1,500-unit vial of anti-D immunoglobulin, BNF [9]
RBC transfusion (units)	3	3	First unit: £194 Subsequent units: £166	£520	£520	Based on the assumption that all units transfused in each treatment arm were RBC.[2]	NICE costing statement for blood transfusion (November 2015).[2] Unit cost for RBC obtained from NHSBT 2016/17[12]

Table K Postoperative resource use and costs

Item	Resource Use		Unit Cost	Mean cost per patient		Source
	**Cell salvage** (n=1498)	**Control** (n=1492)		**Cell salvage** (n-=1498)	**Control** (n=1492)	
Inpatient stay (normal days)	3,734.5	3,852	£431.45	£1,074	£1,113	NHS reference costs 2014/15.[13] Weighted average unit cost for elective and non-elective inpatient bed days
Inpatient stay (HLC)	189.5	136	£539 - £848*	£78	£56	NHS reference costs 2015/15[13] National tariff payment system 2016/17[14]
Adverse events	3	0	n/a	n/a	n/a	BCSH guidelines[3] recommend that the transfusion be discontinued in the event of an adverse reaction
Hospital transfer	2	2	£99	£0.13	£0.13	PSSRU 2015[10]
Investigations	6	10	£94 – 138**	£0.42	£0.70	NHS reference costs 2014/15[13]
Additional surgery	11	8	£399 - £2,991***	£13	£9	NHS reference costs 2014/15[13]
RBC transfusion (units)	3	3	First unit: £190<br>Subsequent units: £165	£13	£17	NICE costing statement for blood transfusion (November 2015).[2] Unit cost for RBC obtained from NHSBT 2016/17[12]
Total cost of postnatal care per patient				£1,178.55	£1,195.83	

* Range based on cost per day of care: Level 1 £539, Level 2 £674. Based on the assumption that level 1 care was 25% more expensive per day than level 0 care and level 2 care was 25% more expensive than level 1 care. Level 3, £848 based on cost per day in intensive care.

** Range based on unit cost of a CT scan (£94) and an MRI scan (£138)

*** Range based on unit cost of additional surgeries (less cost of days in hospital).

Table L Results for the base-case analysis

Transfusion Strategy	Average cost per patient (£)	Effectiveness Donor Blood Transfusion Avoided	ICER (£)
Standard care intended	1,244	0.965	
Cell salvage intended	1,327	0.975	8,110

4. References

1. UK Cell Salvage Action Group. ICS Technical Factsheet: Swab Washing. 2015.
2. National Institute for Health and Care Excellence. Costing statement: Blood transfusion. Implementing the NICE guideline on blood transfusion (NG24). 2015.
3. Tinegate H, Birchall J, Gray A, et al. Guideline on the investigation and management of acute transfusion reactions. Prepared by the BCSH Blood Transfusion Task Force. Br J Haematol 2012;159:143-53.
4. National Institute for Health and Care Excellence. Guide to the Methods of Technology Appraisal. 2013.
5. Gupta SK. Intention-to-treat concept: A review. Perspectives in Clinical Research 2011;2:109-12.
6. Briggs AH, Gray AM. Handling uncertainty when performing economic evaluation of healthcare interventions. Health Technol Assess 1999;3:1-134.
7. Briggs AH. A Bayesian approach to stochastic cost-effectiveness analysis. Health Econ 1999;8:257-61.
8. NHS Supply Chain Catalogue 2015. (Accessed August 2016, at https://my.supplychain.nhs.uk/Catalogue.)
9. British National Formulary. (Accessed August, 2016, at http://www.evidence.nhs.uk/formulary/bnf/current.)
10. Curtis L. Unit Costs of Health and Social Care 2015. Canterbury: Personal Social Services Research Unit, University of Kent; 2015.
11. Qureshi H, Massey E, Kirwan D, et al. BCSH guideline for the use of anti-D immunoglobulin for the prevention of haemolytic disease of the fetus and newborn. Transfusion Medicine 2014;24:8-20.
12. NHS Blood and Transplant. Blood and Components Price List 2016/2017. 2016.
13. Department of Health. NHS Reference Costs 2014/20152015.
14. 2016/17 National Prices. 2016. (Accessed August 2016, at https://www.gov.uk/government/publications/national-tariff-payment-system-2014-to-2015.)
