# Supplementary material for: Cell salvage and donor blood transfusion during cesarean section: A pragmatic, multicentre randomised controlled trial (SALVO)
Source: PLoS Med. 2017 Dec 19;14(12):e1002471. doi: 10.1371/journal.pmed.1002471 (PMC5736174; doi:10.1371/journal.pmed.1002471)
Supplement: S1 Text — (PDF) [file pmed.1002471.s002.pdf]

TITLE OF THE PROTOCOL:

A Randomised Controlled Trial of Intra-Operative Cell Salvage during Caesarean Section in Women at Risk of Haemorrhage.

Short title/Acronym: **SALVO - cell SALVage in Obstetrics**

Sponsor: Queen Mary, University of London (QMUL)

Representative of the Sponsor:
Gerry Leonard
Head of Resources
Joint R&D Office
5 Walden Street
London
E1 2EF
Phone: 020 7882 7260
Email: sponsorsrep@bartshealth.nhs.uk

REC reference: 12/NW/0513

Chief Investigator:

Khalid Khan
Professor of Women's Health and Clinical Epidemiology
Women's Health Research Unit, Blizard Institute
Bart's and The London School of Medicine
Queen Mary, University of London
58 Turner St, London E1 2AB
+44 207 882 2621
k.s.khan@qmul.ac.uk

Trial Sites and Principal Investigators Contact Details

Lead Centre
Barts Health NHS Trust, Royal London Hospital
Dr Matthew Hogg MB, ChB, MRCOG
Consultant in Obstetrics and Gynaecology (obstetric lead),
Women's Centre, Royal London Hospital, Whitechapel, London E1 1BB
07968790384
matthew.hogg@bartsandthelondon.nhs.uk

Birmingham Women's NHS Foundation Trust
Dr James Geoghegan MB, ChB, FRCA
Consultant Anaesthetist
Dept of Anaesthesia, Queen Elizabeth Hospital Birmingham, University Hospitals Birmingham NHS Foundation Trust, Mindelsohn Way, Edgbaston B15 2TH
0121 3713737
james.geoghegan@nhs.net

Heart of England Foundation Trust
Dr Nicola Osborn MB, ChB, FRCA
Dept of Anaesthetics, Bordesley Green East, Birmingham, B9 5ST
0121 424 3438nicola.osborn@heartofengland.nhs.uk

Newcastle Hospitals NHS Trust
Dr Paul Ayuk
Consultant Obstetrician
Directorate Of Women's Services, Newcastle-Upon-Tyne Hospitals NHS Trust, Royal Victoria Infirmary , Queen Victoria Road, NE1 4LP
0191-282-9325
paul.ayuk@nuth.nhs.uk

Professor SC Robson
Professor of Fetal Medicine, Newcastle University
Institute of Cellular Medicine, 3rd Floor Leech Building, Medical School, Framlington Place, Newcastle upon Tyne NE2 4HH
0191 2824132
s.c.robson@ncl.ac.uk

Nottingham University Hospitals
Dr Lesley Woods
Dept of Anaesthetics, Hucknall Road, Nottingham, NG5 1PD
0115 969 1169 ext 55637
lesley.woods@nuh.nhs.uk

Queen's Hospital
Dr Vinod Patil MBBS FRCA
Consultant Anaesthetist, Anaesthesia Dept, Queens Hospital
Rom Valley Way Romford Essex, RM7 0AG
07859919055
vinodpatil@doctors.org.uk

Royal Hallamshire Hospital, Sheffield
Dr Ian Wrench MBChb, BMedSci, PhD, FRCA
Consultant Anaesthetist
Department of Anaesthesia, C Floor, Outpatient department,
Royal Hallamshire Hospital, Sheffield, S10 2JF
01142712381
Ian.Wrench@sth.nhs.uk

Simpson Centre for Reproductive Health, Edinburgh
Dr. Vicki Clark MBChB, FRCA
Consultant Anaesthetist
Dept of Anaesthesia, Royal Infirmary, Little France Crescent, Edinburgh EH16 4SA
01312423151 / 07883568872
vickiaclark@yahoo.co.uk;
vicki.clark@luht.scot.nhs.uk

Singleton Hospital Swansea
Dr Sue Catling BA (Cantab.) MB BS, FRCA
Consultant Obstetric Anaesthetist
Dept Anaesthesia, Singleton Hospital, Sketty Lane, Swansea SA2 8QA
01792 285427 / 07967097411
sue.catling@btopenworld.com

Whipps Cross University Hospital
Dr Sajith Philip
Anaesthetics Dept, Whipps Cross Road, London, E11 1NR
020 8535 6614
Sajith.Philip@bartshealth.nhs.uk

Sunderland Royal Hospital
Dr Aarti Ullal, MRCOG
Sunderland Royal Hospital, Kayll Road, Sunderland Royal Hospital SR4 7TP
0191 541 0051
Aarti.Ullal@chsft.nhs.uk

Royal United Hospital
Dr Chris Marsh MBChB, FRCA
Department of Anaesthesia, Royal United Hospital Bath, Combe Park, Bath, BA1 3NG
07545444078
drcjmarsh@gmail.com

Leicester University Hospitals
Dr Hatem Mousa (Tommy), MD,MD, MRCOG, MSc, MBBCh
University Department of Obstetric and Gynaecology, Leicester Royal Infirmary, Kensington Building, Leicester, LE1 5WW
01162587770
tommy.mousa@uhl-tr.nhs.uk

West Middlesex University Hospital
Dr Dominika Dabrowska, MD
West Middlesex University Hospital NHS Trust, Twickenham Road, Isleworth, TW7 6AF
02083215824
Dominika.Dabrowska@wmuh.nhs.uk

Derriford Hospital
Dr Darryl Thorp-Jones, BSc, MBBS, FRCA
Anaesthetics Department, Lvl 9, Terrence Lewis Building, Derriford Hospital, Derriford Road, Plymouth, PL6 8DH
01752439205
dthorp-jones@nhs.net

University Hospital of North Staffordshire
Dr Jules Allt, MBChB, FRCA
Consultant Anaesthetist and Lead Obstetric Anaesthetist, Department of Anaesthesia and Intensive Care Medicine, University Hospital of North Staffordshire NHS Trust, Newcastle Road, Staffordshire, ST4 6QG
01782 679240
Jules.Allt@uhns.nhs.uk

Torbay Hospital
Dr David Portch, MBChB, FRCA
Anaesthetic Dept, South Devon Healthcare NHS Foundation Trust, Torbay Hospital, Lowes Bridge, Torquay, TQ2 7AA
01803 654311
davidportch@nhs.net

Croydon University Hospital
Dr Bini Ajay, MBBS, MRCOG,MFSRH
Consultant Obstetrician and Gynaecologist, Labour Ward Lead, Department of obstetrics and Gynaecology, Croydon University Hospital, Croydon, CR7 7YE
Tel: 0208 401 3000 x 5905
Bini.Ajay@croydonhealth.nhs.uk
Bini.Ajay@nhs.net

Hinchingbrooke Hospital
Dr Sangeeta Pathak, MBBS, M.Med, DFFP, MRCOG, MD
Consultant Obstetrician, Hinchingbrooke Hospital, Huntingdon, PE29 6NT
01480 442871
sangeetapathak@nhs.net

James Cook University Hospital
Dr Sanjay Rao, MD,MRCOG,MRCPI,DNBE,Dip ClinEd
Consultant Obstetrician and Gynaecologist, The James Cook University Hospital, South Tees NHS Foundation Trust, Marton Road, Middlesbrough, TS4 3BW
01642850850
sanjay.rao@stees.nhs.uk

Northwick Park Hospital
Dr Parijat Bhattacharjee, MRCOG
Consultant Obstetrics & Gynaecology, North West London Hospitals NHS Trust, Watford Road, Harrow, HA1 3UJ
07711874530
parijatbhattacharjee@nhs.net

University Hospitals Bristol, NHS Foundation Trust
Dr Isobel Gardner, MBChB, FRCA
Consultant Anaesthetist, Department of Anaesthesia, St Michaels Hospital, Bristol, BS2 8EG
0117 3425203
Issie.gardner@uhbristol.nhs.uk

Pragmatic Clinical Trials Unit

Blizard Institute, Centre for Primary Care and Public Health
Bart's and The London School Medicine and Dentistry
Queen Mary University of London
Yvonne Carter Building, 58 Turner St
Whitechapel, E1 2AB

PCTU Unit Manager:
Ms Natasha Stevens
+44 207 882 3449
n.stevens@qmul.ac.uk

PCTU Senior Trial statistician:
Richard Hooper
+44 207 882 7324
r.l.hooper@qmul.ac.uk

PCTU Data Manager:
Ms Sandy Smith
+44 207 882 2557
s.l.smith@qmul.ac.uk

PCTU QA Manager: Ms Anitha Manivannan
+44 207 882 6312
a.manivannan@qmul.ac.uk

Protocol development

Dr Philip Moore MBChB MD FRCA FFPMRCA
Consultant Anaesthetist & Honorary Senior Lecturer
Birmingham Women's NHS Foundation Trust
Mindelsohn Way, Edgbaston, Birmingham, B15 2TG
+44 121 472 1377
phil.moore@nhs.net

Dr Matthew Wilson MA (Oxon) BM ChB MD FRCA
Consultant Anaesthetist
Sheffield Teaching Hospitals NHS Trust
Royal Hallamshire Hospital
Glossop Road, Sheffield, S10 2JF
wilsonmja@gmail.com

Professor Khalid Khan
Chief Investigator
+44 207 882 2621
k.s.khan@qmul.ac.uk

Natasha Stevens
PCTU Senior Trial Manager:
+44 207 882 3449
n.stevens@qmul.ac.uk

Richard Hooper
PCTU Senior Trial statistician
+44 207 882 7324
r.l.hooper@qmul.ac.uk

Angela Devine
PCTU Trial Health Economist
+44 207 882 2548
a.devine@qmul.ac.uk

Jane Daniels
Trial Manager
Birmingham Women's NHS Foundation Trust
Mindelsohn Way, Edgbaston, Birmingham, B15 2TG
J.P.Daniels@bham.ac.uk

Chief Investigator Agreement Page

The clinical study as detailed within this research protocol **(Version 6.0, dated 12 September 2014)**, or any subsequent amendments will be conducted in accordance with the Research Governance Framework for Health & Social Care (2005), the World Medical Association Declaration of Helsinki (1996) and the current applicable regulatory requirements and any subsequent amendments of the appropriate regulations.

Chief Investigator Name:

Chief Investigator Site:

Signature and Date:

Statistician Agreement Page

The clinical study as detailed within this research protocol **(Version 6.0, dated 12 September 2014)**, or any subsequent amendments will be conducted in accordance with the Research Governance Framework for Health & Social Care (2005), the World Medical Association Declaration of Helsinki (1996) and the current applicable regulatory requirements and any subsequent amendments of the appropriate regulations.

Statistician Name:

Site:

Signature and Date:

Principal Investigator Agreement Page

The clinical study as detailed within this research protocol **(Version 6.0, dated 12 September 2014)**, or any subsequent amendments will be conducted in accordance with the Research Governance Framework for Health & Social Care (2005), the World Medical Association Declaration of Helsinki (1996) and the current applicable regulatory requirements and any subsequent amendments of the appropriate regulations.

Principal Investigator Name:

Principal Investigator Site:

Signature and Date:

TABLE 1: STUDY SUMMARY/SYNOPSIS

**TITLE**	A Randomised Controlled Trial of Intra-Operative Cell Salvage during Caesarean Section in Women at Risk of Haemorrhage.
**SHORT TITLE**	cell SALVage in Obstetrics – SALVO
**Protocol Version Number and Date**	Version 6.0 dated 12 September 2014
**Methodology**	Individually randomised, controlled, multi-centre study with cost effectiveness analysis.
**Study Duration**	2 years recruitment<br>6 months set up and 6 months data analysis<br>**3 years total**
**Study Centre**	At least Seventeen large obstetric units in collaboration with the Pragmatic Clinical Trials Unit (PCTU).
**Objectives**	Primary Objective:<br>• To determine if the routine use of IOCS during CS, in women at risk of haemorrhage, reduces the need for donor blood transfusion in comparison to current practice.<br>Secondary Objectives:<br>• To determine the effect of IOCS on secondary outcomes including the number of units of donor blood transfused, mean fall in serum haemoglobin level and maternal morbidity resulting from post-operative anaemia (time to first mobilisation, duration of hospital stay, and immediate multidimensional fatigue inventory.<br>• To determine if the routine use of IOCS during CS, in women at risk of haemorrhage, is cost effective in comparison to current practice.
**Number of Participants**	3050
**Eligibility Criteria**	**Inclusion Criteria**<br>Women who are admitted to a participating labour ward who fulfil all the following criteria will be eligible to be randomised:<br>• 16 years of age or older<br>• Delivery by elective or emergency caesarean section with an identifiable increased risk of haemorrhage.<br>• Ability to provide informed consent<br>**Exclusion Criteria**<br>• Elective first Caesarean section for maternal request or breech presentation, with no additional prognostic factor for haemorrhage<br>• Sickle cell disease<br>• Active malignancy contraindicated to CS e.g. abdominal cancer<br>• Cultural or social beliefs contraindicating blood transfusion e.g. Jehovah's Witnesses.<br>• Significant antibodies making it difficult to find cross matched blood for transfusion<br>• Unable to understand written and spoken English
**Statistical Methodology and Analysis**	A detailed analysis plan will be developed and agreed by the Trial Steering Committee and the Data Monitoring Committee, prior to unblinding and data analysis. Demographic factors and clinical characteristics will be summarised with counts (percentages) for categorical variables, mean (standard deviation[SD]) for normally distributed continuous variables, or median (interquartile [IQR] or entire range) for other continuous variables. The primary analysis will be a comparison of the management policies assigned at randomisation (intention-to-treat). The risk of the primary outcome in the IOCS group will be compared with the usual practice group and tested for significance at the two-sided 5% level of significance. Crude and adjusted odds ratios and 95% confidence intervals will be produced. Adjusted analyses will allow for known or suspected prognostic factors, to be specified in the analysis plan. Analysis of secondary outcomes will be clearly delineated from the primary analysis in any statistical reports produced.

Glossary of Terms and Abbreviations

AAGBI	Association of Anaesthetists of Great Britain and Ireland
AE	Adverse Event
AR	Adverse Reaction
ASA	American Society of Anesthesiologists
ASR	Annual Safety Report
CA	Competent Authority
CEMACE	Centre for Maternal and Child Enquiries
CEMACH	Confidential Enquiry into Maternal and Child Health
CI	Chief Investigator
CLRN	Comprehensive Local Research Network
CRF	Case Report Form
CRO	Contract Research Organisation
CS	Caesarean Section
DMC	Data Monitoring Committee
EC	European Commission
GAfREC	Governance Arrangements for NHS Research Ethics Committees
ICF	Informed Consent Form
IOCS	Intra-Operative Cell Salvage
ISRCTN	International Standard Randomised Controlled Trial Number
MA	Marketing Authorisation
MS	Member State
MREC	Main Research Ethics Committee
NHS R&D	National Health Service Research & Development
NIHR CRN	National Institute for Health Research - Clinical Research Network
NICE	National Institute of Clinical Excellence
OAA	Obstetric Anaesthetists Association
Participant	An individual who takes part in a clinical trial
PI	Principle Investigator
PCTU	Pragmatic Clinical Trials Unit
QA	Quality Assurance
QC	Quality Control
RCT	Randomised Controlled Trial
REC	Research Ethics Committee
RCOG	Royal College of Obstetricians and Gynaecologists
SAE	Serious Adverse Event
SDV	Source Document Verification
SHOT	Serious Hazard of Transfusion
SOP	Standard Operating Procedure
SSA	Site Specific Assessment
TMG	Trial Management Group
TSC	Trial Steering Committee
UK	United Kingdom
UKCRN	United Kingdom Clinical Research Network
UKSAG	UK Cell Salvage Action Group

Table of Contents

TRIAL SITES AND PRINCIPAL INVESTIGATORS CONTACT DETAILS 2

PROTOCOL DEVELOPMENT 5

CHIEF INVESTIGATOR AGREEMENT PAGE 7

STATISTICIAN AGREEMENT PAGE 8

PRINCIPAL INVESTIGATOR AGREEMENT PAGE 9

TABLE 1: STUDY SUMMARY/SYNOPSIS 10

GLOSSARY OF TERMS AND ABBREVIATIONS 11

1 INTRODUCTION 15

1.1 BACKGROUND 15
1.2 CLINICAL DATA 15
1.2.1 CLINICAL BACKGROUND 15
1.2.2 CELL SALVAGE IN CAESAREAN SECTION 16
1.3 RATIONALE AND RISKS/BENEFITS 16
1.3.1 RISKS 16
1.3.2 BENEFITS 17
1.3.3 CURRENT POLICY AND PRACTICE 17
1.3.4 WORK LEADING TO THE PROPOSAL 17
1.3.5 SYSTEMATIC REVIEW OF THE LITERATURE 18
1.3.6 FEASIBILITY OF UNDERTAKING THE TRIAL – PILOT STUDY 18
1.3.7 SURVEY OF WOMEN DELIVERED BY CAESAREAN SECTION 18
1.3.8 SURVEY OF CLINICIANS 19
1.3.9 AUDIT OF TRANSFUSION RATES IN CAESAREAN SECTION 20

2 TRIAL OBJECTIVES AND DESIGN 20

2.1 TRIAL OBJECTIVES 20
2.1.1 PRIMARY OBJECTIVE: 20
2.1.2 SECONDARY OBJECTIVES: 20
2.2 TRIAL DESIGN 20
2.3 SETTING 20

3 SUBJECT SELECTION 22

3.1 FIGURE 3: ANTICIPATED STUDY CONSORT DIAGRAM 22
3.2 ELIGIBILITY CRITERIA 23
3.2.1 INCLUSION CRITERIA 23
3.2.2 EXCLUSION CRITERIA 23
3.3 PROTOCOL VIOLATIONS 23
3.4 DATA COLLECTION AND FOLLOW UP FOR WITHDRAWING PARTICIPANTS 23

4 STUDY PROCEDURES 24

4.1. INFORMED CONSENT PROCEDURES 24
4.1.1. ELECTIVE CAESAREAN SECTION 25
4.1.2. EMERGENCY CAESAREAN SECTION 25
4.2. SCREENING PROCEDURES 28

4.3. RANDOMISATION PROCEDURES 28
4.4. METHODS FOR MINIMISING THE RISK OF BIAS 28
4.5. PREPARING AND SUPPORTING THEATRE AND MEDICAL STAFF FOR PARTICIPATION IN THE TRIAL 29
4.6. SCHEDULE OF TREATMENT 29
4.7. TABLE 3: SCHEDULE OF ASSESSMENT 30
4.8. CRITERIA FOR EARLY TERMINATION OF THE STUDY 31
4.9. END OF STUDY DEFINITION 31

5. LABORATORIES (IF APPLICABLE) 31

5.1. CENTRAL/LOCAL LABORATORIES 31
5.2. SAMPLE COLLECTION/LABELLING/LOGGING 31
5.3. SAMPLE RECEIPT/CHAIN OF CUSTODY/ACCOUNTABILITY 31
5.4. SAMPLE ANALYSIS PROCEDURES 31
5.5. SAMPLE STORAGE PROCEDURES (IF APPLICABLE) 31
5.6. DATA RECORDING/REPORTING 31

6. PHARMACOVIGILANCE 32

6.1. GENERAL DEFINITIONS 32
6.1.1. ADVERSE EVENT (AE) 32
6.1.2. SERIOUS ADVERSE EVENT (SAE) 32
6.2. INVESTIGATORS ASSESSMENT 32
6.2.1. SERIOUSNESS 32
6.2.2. CAUSALITY 32
6.2.3. EXPECTEDNESS 32
6.2.4. SEVERITY 32
6.3. NOTIFICATION AND REPORTING ADVERSE EVENTS OR REACTIONS 33
6.4. NOTIFICATION AND REPORTING OF SERIOUS ADVERSE EVENTS 33
6.4.1. EXPECTED ADVERSE EVENTS 33
6.5. URGENT SAFETY MEASURES 33
6.6. ANNUAL SAFETY REPORTING 34
6.7. OVERVIEW OF THE SAFETY REPORTING PROCESS/PHARMACOVIGILANCE RESPONSIBILITIES 34

7. STATISTICAL CONSIDERATIONS 34

7.1. TRIAL OUTCOMES 34
7.1.1. PRIMARY OUTCOME 34
7.1.2. SECONDARY OUTCOMES 34
7.1.3. SAFETY OUTCOMES 34
7.1.4. PROCESS OUTCOMES 34
7.2. PRIMARY ANALYSIS 35
7.3. SECONDARY ANALYSIS 35
7.4. SAFETY ENDPOINTS 35
7.5. SAMPLE SIZE 35
7.6. STATISTICAL ANALYSIS 37
7.7. HEALTH ECONOMICS ANALYSIS 38
7.7.1. COST DATA COLLECTION 38
7.7.2. ANALYSIS 38

8. DATA HANDLING & RECORD KEEPING 39

8.1. CONFIDENTIALITY 39
8.2. REQUIRED STUDY DOCUMENTS 39

8.3. CASE REPORT FORMS 40
8.4. DATA COLLECTION, PROCESSING AND MONITORING 41
8.5. CENTRAL STATISTICAL MONITORING 41
8.6. RECORD RETENTION AND ARCHIVING 41
8.7. QUALITY CONTROL AND QUALITY ASSURANCE 41
8.7.1. SUMMARY MONITORING PLAN 41
8.7.2. AUDIT AND INSPECTION 42
8.7.3. COMPLIANCE 42
8.7.4. NON-COMPLIANCE 42

9. CLINICAL GOVERNANCE ISSUES 43

9.1.1. ETHICAL CONSIDERATIONS 43
4.1.1 NETWORK COLLABORATION 43
9.1.2. NATIONAL REGISTRATION SYSTEMS 43
9.1.3. FUNDING AND FINANCIAL ASPECTS OF THE TRIAL 43

10. TRIAL COMMITTEES 43

10.1. CLINICAL INVESTIGATORS GROUP 43
10.2. LOCAL CO-ORDINATION 44
10.3. TRIAL MANAGEMENT GROUP 44
10.3.1. SITE SET-UP AND TRAINING 44
10.3.2. PROJECT TIMETABLE, MILESTONES AND PROJECTED RECRUITMENT 44
10.4. TRIAL STEERING COMMITTEE 47
10.5. CONSUMER REPRESENTATION 48
10.6. DATA MONITORING COMMITTEE 48

11. PUBLICATION POLICY 49

12. REFERENCES 50

APPENDIX 1 – COMMUNICATION ORGANOGRAM FOR REPORTING SAE'S 52

APPENDIX 2 – INFORMATION WITH REGARDS TO SAFETY REPORTING IN NON-CTIMP RESEARCH 53

APPENDIX 3: REQUIREMENT FOR BLOOD TRANSFUSION FLOW DIAGRAM 54

APPENDIX 4: SITE CODES 55

1 Introduction

1.1 Background

Haemorrhage (excessive blood loss) remains the leading cause of direct maternal death in sequential Confidential Enquiry into Maternal and Child Health (CEMACE) reports. Haemorrhage is more common in women who have Caesarean sections, whether planned Caesarean sections for complications such as placenta previae (low lying placenta) or emergency Caesarean sections when the baby is in distress. Approximately 70,000 units of blood (known as Packed Red Cells) are given annually in the maternity setting at a current cost of £140 per unit. Approximately 140,000 Caesarean sections (CS) are performed annually in the UK, so any reduction in the amount of blood required could significantly reduce the cost of blood transfusions to the NHS

Blood is a scarce and expensive resource for the NHS and the availability of blood for transfusion is likely to decrease. Its availability is an essential prerequisite for major procedures including joint replacement, cardiac surgery, organ transplantation, cancer care and the management of trauma. Its scarcity places constant limitations on the ability of the NHS to deliver high quality health care to all points of need simultaneously. Priority decisions result in the deferment or cancellation of procedures if this resource is required elsewhere. Because of the scarcity of blood and the concerns about possible risks to the patient of giving them a blood transfusion; as a general rule, obstetric patients are often allowed to become relatively anaemic (low blood haemoglobin levels) post operatively. Post-operative anaemia has been associated with a longer hospital stay, increased wound infection rates and delayed time to mobility.

Intra-operative cell salvage (IOCS) collects the patient's own blood lost during an operation, processes it and returns it to their circulation. This may lead to less postoperative anaemia which may in turn reduce morbidity and hospital stay. Its use has been proven to reduce the amount of donor blood given in other operations, however its use in CS has not yet been adequately examined, due in part to concerns about contamination of salvaged blood with amniotic fluid, which have proven unfounded (10;11). Donor blood transfusion carries with it significant risks, which IOCS has the potential to avoid, including cross-matching errors, infection and transfusion reaction. The National Institute of Clinical Excellence (NICE) currently only recommends IOCS for massive blood loss in emergency CS, but has called for robust evidence from clinical trials to support its wider, routine use (12).

1.2 Clinical Data

1.2.1 Clinical Background

A series of tri-annual Confidential Enquiry into Maternal and Child Health reports, including the latest, have consistently identified haemorrhage as an important direct cause of maternal death (13). Life threatening blood loss is the primary indication for 95.6% of emergency hysterectomies in labour (14). Haemorrhage is the commonest cause for maternal critical care admission (15;16) and places a profound health burden on the childbearing population during an important life event. Caesarean section accounts for 24.6% of deliveries in the UK (2008-9). It is the commonest operation conducted by the NHS with over 400 performed per day in England alone. Major haemorrhage can occur without warning during CS and quickly overwhelm attempts to correct blood loss. The likelihood of haemorrhage is increased by risk factors including previous CS, morbidly adherent placenta, emergency CS for any indication, ante-partum haemorrhage and pre-eclampsia. The principle treatment for major haemorrhage is allogeneic (donor) blood transfusion. Approximately 200 units of blood are given each day for obstetric emergencies. At £140 per unit, this equates to £10.2M per year without considering the financial consequences of maternal acute illness. Donor blood is a finite, expensive, nationally pooled resource. Shortages of donor blood are increasingly common. All NHS

hospitals are required to have policies for blood shortages, including cancellation of elective surgery which may require transfusion. There are major risks associated with donor blood transfusion, including death from transfusion error, acute transfusion reaction, fatal lung injury and infection transmission (17). These risks are monitored by annual Serious Hazards of Transfusion (SHOT) reports (18). Despite improved safety mechanisms, they show little sign of diminishing. Donor blood is therefore used judiciously in the healthy obstetric population. This high transfusion threshold can result in post-natal maternal anaemia and associated morbidity, including maternal fatigue, an increased rate of wound infection and delayed mobility after childbirth. Anaemia prolongs hospital stay by a third, with an overall 50% higher cost per hospitalization (19). The economic consequences of anaemia resulting from obstetric haemorrhage are therefore profound and any intervention which could reduce maternal morbidity and mortality is worthy of scrutiny.

A technology that simultaneously reduces the need for donor blood transfusion and prevents anaemia could avoid the serious morbidity associated with haemorrhage. Intra-operative cell salvage has been proven to achieve precisely this in non-obstetric surgery with a significant reduction in operative costs. Whether IOCS is similarly effective in CS requires rigorous examination. We propose a randomised controlled trial with health economic evaluation to perform a technology assessment.

1.2.2 Cell salvage in Caesarean section

IOCS collects blood lost by a patient during surgery and returns it to their circulation. In recent years the technology has been refined and has entered routine use in cardiac, orthopaedic, liver and vascular surgery where there is a risk of major haemorrhage. National guidelines only currently recommend IOCS in obstetrics in the emergency management of massive haemorrhage. Moderate blood loss is a normal expectation during uncomplicated CS. By salvaging this blood, it may be returned to the patient, even when donor blood transfusion would not normally be considered. This might reduce post-operative anaemia and its associated morbidity. IOCS is beginning to enter routine use in CS in some centres, with the aim of realising some of these benefits. However, use in this context remains unproven and is not supported by evidence for its clinical or economic effectiveness. IOCS has the potential for significant cost savings to the NHS compared with current practice. CS is a very common operation and the cost per patient of routine IOCS is approximately the same as a single unit of blood. This must be set against the cost of blood transfusion, the care costs of prolonged hospital stay and the expense of treating adverse events associated with transfusion. IOCS could realise the dual economic goals of earlier hospital discharge and enhanced quality of life.

1.3 Rationale and Risks/Benefits

1.3.1 Risks

Previously the obstetric setting was a contraindication to the use of IOCS because of theoretical concerns regarding the risk of amniotic fluid embolus, a serious but extremely rare (about 1 in 20,000) complication of pregnancy/childbirth. As our understanding of this condition has improved, the theoretical risk has greatly reduced. Studies examining the quality of blood that would be returned to the mother, had IOCS been used at caesarean section, have shown that, with modern equipment, there is no difference between blood processed at caesarean section and returned to the mother and normal maternal blood (10;11).

Another potential risk associated with IOCS is “Rhesus sensitization”. Rhesus disease occurs when there is an incompatibility between antibodies carried on red blood cells of a woman and her infant e.g. a rhesus-negative (Rh-) woman giving birth to a baby with a rhesus-positive (Rh+) blood type. Contact between maternal and fetal blood may provoke an immune response in the maternal immune system. The antibodies produced can induce fetal haemolytic disease in future pregnancies. There is no evidence to suggest that IOCS increases the risk of sensitization.

Maternal exposure to fetal blood is measured using a Kleihauer test. All rhesus-negative women delivering by Caesarean section receive anti-D antibody injection to prevent sensitization. This risk is therefore not appreciably different between women in the IOCS or standard care groups. The National

Institute for Health and Clinical Excellence (NICE) has issued guidelines regarding the use of IOCS in obstetrics. They state that the technology may be of benefit with careful patient selection. In addition to NICE, IOCS in obstetrics is also recommended by CEMACH (Confidential Enquiry into Maternal and Child Health), the RCOG (Royal College of Obstetricians and Gynaecologists), the OAA (Obstetric Anaesthetists Association) and the AAGBI (Association of Anaesthetists of Great Britain and Ireland) in the UK and the ASA (American Society of Anesthesiologists) in the USA.

The use of leukocyte depletion filters in the return of salvaged blood has been the subject of scrutiny in the medical literature. There are some reports of unexplained hypotension associated with blood return and filters have been implicated as a potential source of anaphalactoid response. Whilst inclusion of a filter is currently recommended, they may restrict rapid re-infusion of blood in the context of massive haemorrhage and are routinely omitted at the discretion of clinicians when rapid blood return is imperative. We will monitor any reports of severe, unanticipated hypotension and their potential association with the presence of leukocyte filters to inform this on-going debate.

Risks associated with donor blood transfusion include death from transfusion error, acute transfusion reaction, fatal lung injury and infection transmission.

It is not anticipated that there be any additional pain, discomfort, distress, inconvenience or changes to lifestyle for research participants receiving IOCS compared to the standard care group. It is not anticipated that there will be any potential for adverse events, risks or hazards, pain, discomfort, distress, or inconvenience to the researchers themselves.

1.3.2 Benefits

IOCS may reduce the need for a standard donor blood transfusion and consequently, there should be fewer transfusion reactions and infections as the blood is known to be the correct type for the patient. Post-operative anaemia has been associated with a longer hospital stay, increased wound infection rates, and delayed time to mobility. IOCS allows re-transfusion of the patient's own blood that would otherwise have been wasted. This may lead to less postoperative anaemia, which may in turn reduce morbidity and hospital stay.

1.3.3 Current policy and practice

The National Institute for Health and Clinical Excellence (NICE) has issued guidelines regarding the use of IOCS in obstetrics. They state that the technology may be of benefit with careful patient selection. In addition to NICE, IOCS in obstetrics is also recommended by CEMACH (Confidential Enquiry into Maternal and Child Health), the RCOG (Royal College of Obstetricians and Gynaecologists), the OAA (Obstetric Anaesthetists Association) and the AAGBI (Association of Anaesthetists of Great Britain and Ireland) in the UK and the ASA (American Society of Anesthesiologists) in the USA.

A national survey 2005-6 reported that 38% of UK maternity units had access to IOCS and 12% included it in their major obstetric haemorrhage protocol (20). The national uptake of IOCS in obstetrics is not based on the evidence (see work leading to the proposal). Opinion, however, is not yet solidified in the clinical community and there is genuine equipoise. There is no better time to launch a large multicentre RCT to generate reliable, valid evidence.

1.3.4 Work leading to the proposal

In preparing this proposal we have undertaken a systematic literature review, performed a pilot randomised controlled trial, surveyed women and clinicians, and audited event rates. This has helped establish the need for the trial, confirmed feasibility and informed trial design.

1.3.5 Systematic review of the literature

We published (1) and updated a systematic review which identified one small controlled trial of IOCS in CS, with 34 participants in each group, which reported a significant reduction in the number of participants requiring transfusion. However, there were flaws in trial design and conduct, including no explanation of the randomisation method. Furthermore, the control group transfusion rate of 23.5% is at least more than three times greater than normal practice in the UK. The methodology employed in other studies, including retrospective review, two case series and isolated case reports, preclude definitive conclusions but support the safety of IOCS in obstetrics. The adoption of IOCS into obstetric practice was limited by concerns about the potential risk of contamination of blood salvaged at CS with amniotic fluid. However, the risk of amniotic fluid embolus has proven theoretical rather than actual. Studies examining the components of blood recovered with modern salvage equipment have demonstrated that every element of amniotic fluid is effectively removed (10;11). The responses to our review supported the need for a definitive trial.

A recent NICE review of IOCS (IPG144 2005) (12) stated that the technology may be of benefit with careful participant selection and that shortage of donor blood supplies makes IOCS a pertinent issue. The review focused on the lack of high quality research and called for randomised controlled trials. The Royal College of Obstetricians and Gynaecologists (RCOG) “Greentop” Guidelines (12/2007) (21) recognised that "cell salvage in obstetrics remains controversial". The evidence is graded C as a result of the absence of trials on which to base recommendations. A Cochrane review and other meta-analyses of the use of IOCS in non-obstetric settings, demonstrated a significant reduction in patient exposure to donor blood (7). A recent HTA report put the relative risk of exposure at 0.59 (95% CI 0.48 to 0.73) for the pooled trials of IOCS (6). Evidence from population studies of women with post-partum anaemia have demonstrated a 33% increase in hospital stay and a 50% increase in costs of hospitalisation (19).

An economic model, drawn from primary cost studies and randomised trials, concluded that IOCS had lower costs and higher quality-adjusted life years compared with all other alternative transfusion strategies except acute normo-volaemic haemodilution (6). However this model did not include CS, limiting generalisability to the obstetric setting.

1.3.6 Feasibility of undertaking the trial – Pilot study

A pilot randomised controlled trial of IOCS in elective (planned) CS in one of the co-applicant centres was approved by the ethics committee and the local trust R&D at Birmingham Women’s Hospital. This study has helped to refine trial policies and practice, tested the utility of data collection methods and acceptability of the study design. At closure, 57 women had been randomised. A confidential interim data analysis performed by Birmingham Clinical Trials Unit (who supported the pilot trial), keeping investigators blind to group allocation, was undertaken for preparing this application. It has shown that:

1. The consent rate was 71% (of the 80 women approached, 23 declined to participate).
2. The primary end point data were collected for 100% of randomised women.
3. The use of IOCS was feasible and acceptable to staff and to women randomised.
4. Blood salvage and return was technically unproblematic requiring minimal additional resource.
5. Adherence to the randomisation strategy was high with 1 case of use of IOCS in the control group (see implication in section 3.5).

1.3.7 Survey of women delivered by Caesarean Section

A survey of women undergoing CS in whom IOCS was used for anticipated haemorrhage was conducted at Sheffield Teaching Hospital, one of the co-applicant centres. The majority of CSs were performed “awake” under regional anaesthesia for enhanced maternal and fetal safety. The opinions of the recipients of IOCS in CS are therefore particularly pertinent to inform any proposed study advocating its routine use.

Women scheduled for CS were given written and verbal information about IOCS prior to operation. A self-administered questionnaire was employed in the immediate postnatal period. Of 71 women surveyed, 69 completed and returned the questionnaire, a response rate of 97%. The responses in Figure 1 confirm that women were reassured by the prospect that IOCS could re-transfuse their own blood and perceived the technology as lower risk than donor blood transfusion. Twenty two women went on to have IOCS with 16 (72%) receiving salvaged blood. Of these 22 women, 17 (77%) expressed a preference for IOCS to be made available in the event of them requiring a CS in the future.

The results of the survey (Figure 1) demonstrate that the attitude to IOCS during CS in women is very positive and that they regard the technology as reassuring and safe.

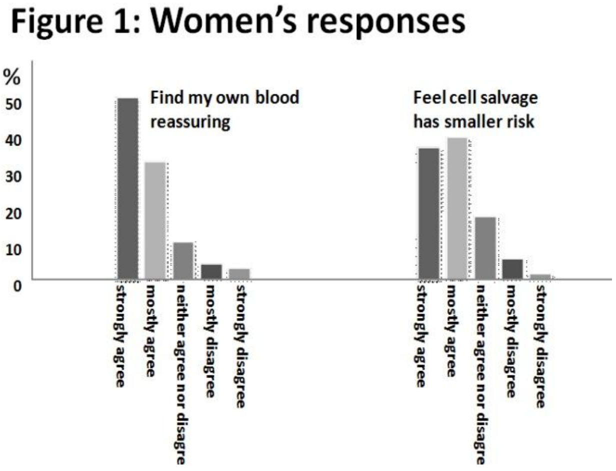

1.3.8 Survey of Clinicians

A separate online survey of lead clinicians in potential recruiting centres was undertaken to estimate the recruitment pool and to assess preparedness to randomise women in certain situations. Of those units responding to the survey, all had IOCS technology available to obstetric cases: 70% of centres had this availability on a 24 hour basis; 20% had availability restricted to daytime "office" hours or more often dependent on the presence of trained staff; and in 10% salvage had to be pre-arranged. Clinicians were also asked to indicate their preparedness to randomise women in specific categories of increased risk of haemorrhage (summarised in figure 2).

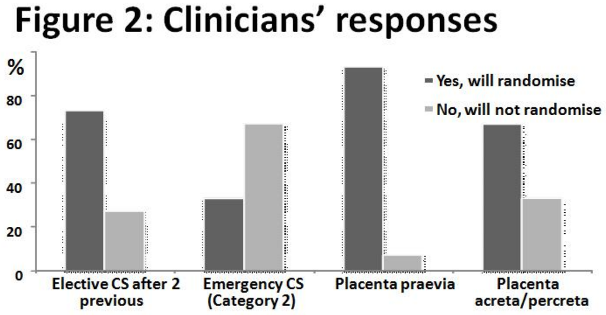

The survey adds substantially to the premise that the study is deliverable and that sufficient equipoise exists in the clinical community to enable rigorous hypothesis testing. It was reassuring that clinicians expressed clear willingness to randomise women at increased haemorrhage risk with 94% responding that they would randomise in the context of placenta praevia; this commitment persisted even in the presence of morbidly adherent placenta, and two previous CS. In the case of emergency CS, with no immediate fetal risk (Category 2), just under 40% of respondents stated that randomisation was

feasible, reflecting the realistic perception that recruitment in labour in the emergency setting is very challenging. These clinical perceptions have been used to inform the projected recruitment.

1.3.9 Audit of transfusion rates in Caesarean Section

A detailed audit of donor blood use at the Royal Hallamshire Hospital Sheffield 2009-10, without IOCS in routine use, was performed by cross-reference of perioperative records, blood bank data and electronic records stored in cell salvage machines. It reported that in a recent series of 1647 CS over 10 months, 89 women were transfused with donor blood, giving a rate of 5.4% (I. Wrench, Personal communication). A similar audit at Birmingham Women's Hospital of all CS carried out in 2006, showed that of 1674 women, 83 (5.0%) received a transfusion, with a mean transfusion volume of 1620ml SD 1350ml (1). This equates to 3.6 +/- 3 units of donor blood administered. Both auditing units deliver approximately 7300 women per year with a similar CS rate and can be considered representative of UK tertiary obstetric unit practice.

It is important to note that these data include both acute transfusion at operation and any donor blood given in the postoperative period to treat symptomatic anaemia before hospital discharge. Our study population is defined by women undergoing CS who have at least one identifiable risk factor for haemorrhage. We have defined 'Increased risk of haemorrhage' to include elective CS for abnormal placentation and previous CS and all emergency CSs. The emergency CS group includes a number of indications which are at particularly high risk of haemorrhage including antepartum haemorrhage and pre-eclampsia. The baseline transfusion requirement of this selected population is likely to be elevated. For example, a recently published study of 246 Caesarean deliveries for placenta praevia reported 29% of cases required transfusion. Of those women who received donor blood, 63% required 3 units or more (23).

2 Trial Objectives and Design

2.1 Trial Objectives

2.1.1 Primary Objective:

To determine if the routine use of IOCS during CS, in women at risk of haemorrhage, reduces the need for donor blood transfusion in comparison to current practice.

2.1.2 Secondary Objectives:

- To determine the effect of IOCS on secondary outcomes including the number of units of donor blood transfused, mean fall in serum haemoglobin level and maternal morbidity resulting from post-operative anaemia (time to first mobilisation, duration of hospital stay, and immediate post natal multidimensional fatigue inventory).
- To determine if the routine use of IOCS during CS, in women at risk of haemorrhage, is cost effective in comparison to current practice.

2.2 Trial Design

A multicentre individual randomised controlled trial with cost-effectiveness analysis.

2.3 Setting

At least seventeen large obstetric units supported by the Pragmatic Clinical Trials Unit (PCTU). The following table (Table 2) gives demographic details for proposed units (29):

Table 2: Proposed Trial Hospital Sites Annual C-sections and Deliveries

Hospital	CLRN	Proposed PI	Annual Deliveries	Annual C-Sections
Royal London Hospital	CEL	Matthew Hogg	4400	1012
Homerton University Hospital	CEL	Sade Okutubo	5000	1300
Queen's Hospital, Romford	CEL	Remi Odejinmi	8000	1840
Whipps Cross University Hospital	CEL	Sadanand Chitre	5500	1375
Guys & St Thomas' Hospitals NHS Trust	London (South)	Geraldine O'Sullivan	7000	1890
John Radcliffe Hospital Oxford	Thames Valley	Robin Russell	7000	1600
Birmingham Women's NHS Foundation Trust	BBC	James Geoghegan	7300	1825
Heart of England Hospital Foundation Trust	BBC	Elizabeth Walker	7000	1610
University Hospitals Coventry & Warwick	West Midlands (South)	John Elton	6000	1560
Central Manchester University Hospital	Greater Manchester	Richard Wadsworth	6400	1216
Royal Hallamshire Hospital, Sheffield	South Yorkshire	Ian Wrench	7500	1725
St James University Hospital Leeds	West Yorkshire	Rowan Wilson	5000	1150
Nottingham City Hospital	Trent	Lesley Woods	5793	1092
Newcastle Hospitals NHS Trust	Northumb, Tyne & Wear	Paul Ayuk	7000	1750
Singleton Hospital Swansea	CRC Cymru	Sue Catling	3800	874
Simpson Centre for Reproductive Health, Edinburgh	CRC Scotland	Vicki Clark	7000	1890
Hull and East Yorkshire NHS Trusts	North East Yorkshire and North Lincolnshire CLRN	Packianathasw-amy Balaji	5661	1313
Chelsea and Westminster Hospital	London NW CLRN	Dr Amer Raza	5646	2023
North Bristol NHS Trust	The Western CLRN	Dr Tim Draycott	6435	1541
**Total per year**			**117, 435**	**28, 586**

3 Subject Selection

3.1 Figure 3: Anticipated Study Consort Diagram

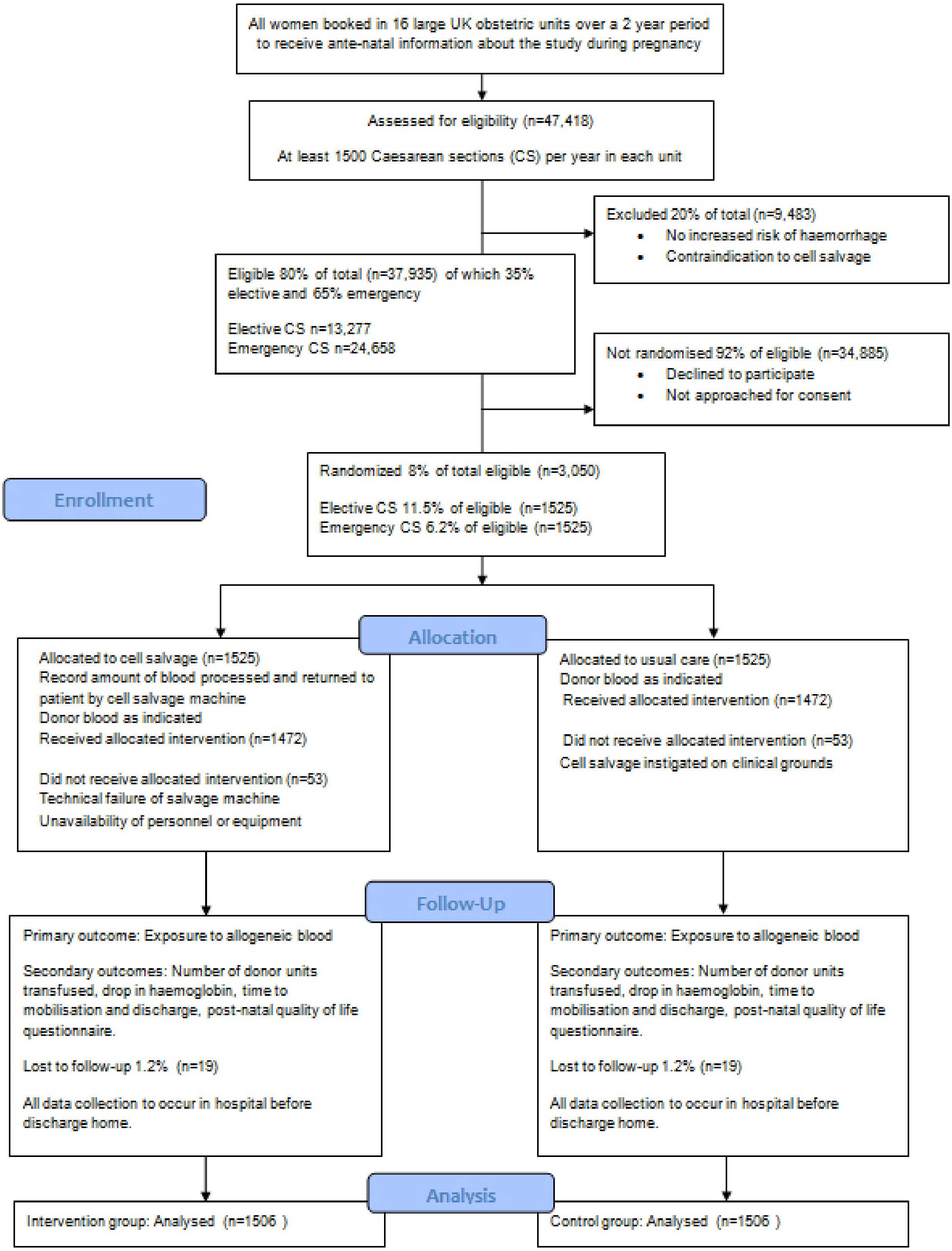

3.2 Eligibility Criteria

Women who are admitted to a participating labour ward who fulfil all the following criteria will be eligible to be randomised.

3.2.1 Inclusion Criteria

- 16 years of age or older
- Delivery by elective or emergency caesarean section with an identifiable increased risk of haemorrhage.
- Ability to provide informed consent

3.2.2 Exclusion Criteria

- Elective first Caesarean section for maternal request or breech presentation; with no additional prognostic factor for haemorrhage
- Sickle cell disease
- Active malignancy contraindicated to CS e.g. abdominal cancer
- Cultural or social beliefs contraindicating blood transfusion e.g. Jehovah's Witnesses.
- Significant antibodies making it difficult to find cross matched blood for transfusion
- Unable to understand written and spoken English

The exclusion criteria above have been chosen for the following reasons;

a. Elective first CS due to maternal request or breech presentation does not tend to put the mother at an increased risk of haemorrhage. All other indications for CS would be considered an identifiable increased risk of haemorrhage.
b. Significant antibodies make it difficult to find cross matched blood because allogeneic blood for this group of patients is likely to be scarce or unavailable. We consider it appropriate to give these patients IOCS from the start of their case.
c. Since donor red blood cell transfusion is the primary study outcome, individuals with cultural or social beliefs that preclude donor blood transfusion will be excluded from the study.
d. Women with sickle cell disease have low levels of oxygen, this can cause the red blood cells to deform and block the microscopic blood vessels in the body. There is a chance that this "sickling" may occur while the blood is in the IOCS collection reservoir awaiting processing.
e. Women with active cancer contraindicated to CS, especially cancer in the abdominal region, will be excluded as there is a theoretical risk of spreading the cancer should IOCS be used.

3.3 Protocol violations

A participant will be considered a protocol violator if, after randomisation:

e.g.

- They no longer meet eligibility criteria
- Due to safety concerns for the participant, or at investigator discretion, the opposite intervention (i.e. IOCS in the case of randomisation to routine practice) is definitely indicated
- Participant loses capacity to consent
- Participant withdraws their consent, to either allocated treatment and/ or to data collection.
- Participant no longer requires a caesarean section

3.4 Data Collection and Follow up for Withdrawing Participants

A participant can be withdrawn from the trial treatment if, in the opinion of the investigator or the care providing clinician or clinical team, it is medically necessary to do so. Withdrawal from follow-up is the decision of the participant. However, withdrawn participants can bias clinical trial results

and reduce the power of the trial to detect important differences. With any protocol violation, the study personnel will make every effort to obtain, and record, information about the reasons for violation, any adverse events and to follow-up the women for all safety and efficacy outcomes, as appropriate. If a woman decides after randomisation she does not wish to participate any further in the SALVO trial, she may withdraw herself from the trial. We will aim to document the reason for self-withdrawal. Clear distinction will be made as to whether the participant is withdrawing from trial whilst allowing further follow-up, or whether the participant refuses any follow-up. If a participant explicitly withdraws consent to have any further data recorded their decision will be respected and recorded on the final study form. All communication surrounding the withdrawal will be noted in the study records and no further data will be collected for that participant. They will be returned to the NHS standard practice for follow up care.

If a woman loses the capacity to consent during participation in the trial, they will be withdrawn from the trial and no further data will be collected from the participant unless consent for this was explicitly obtained prior to the loss of capacity.

Participants who are protocol violators will not be replaced by another participant as this has been accounted for in the sample size calculation and estimated at 1.2%.

3.5 Choice of outcome measure

The primary outcome will be use of donor blood transfusion. Reducing the proportion of women with this outcome should lead to fewer transfusion related complications. The treatment arms will be compared according to this outcome on an intention-to-treat basis. However, because clinicians managing women in the control arm will have access to an IOCS machine, it is possible that women in the control arm could receive IOCS in place of a donor blood transfusion. As a sensitivity analysis, we will analyse the primary outcome assuming that all instances of the use of IOCS in the control arm would have been instances of donor blood transfusion had the IOCS machine not been present. We will aim to minimise the sensitivity of the estimate of treatment effect to this assumption by promoting equipoise among participating clinicians and emphasising the importance of adherence to the protocol.

For the avoidance of doubt it is important to clarify terminology. Sometimes use of an intervention in the control group for whom it is not intended is described as ‘crossover’. This can be confusing as crossover trial is a specific trial design that we are not employing in our project. We have therefore refrained from the use of the term ‘crossover’ throughout this protocol.

4 Study Procedures

4.1 Informed Consent Procedures

As many women as possible booked to deliver at participating centres, whether they are intending a natural (vaginal) delivery or a caesarean section, will receive information about the study during their pregnancy and again on admission to delivery suite. This process will be individualised for each participating centre depending on their routine practice to ensure that the maximum number of women are offered information well in advance of delivery. For example, in some centres, women will be provided with information about the trial at their routine anomaly scan appointment (18-22 weeks). The provision of study information will be documented in the woman’s medical record or handheld notes, and a sticker applied to indicate whether they are or are not interested in taking part in the study, or whether they have not yet made up their mind. Also it will be further documented at this point if in an emergency situation they would still be interested in taking part in the study.

Written informed consent will be obtained by a health professional (obstetrician, anaesthetist or midwife) with delegated authority from the Principal Investigator. All women will be assessed to ensure that they have the capacity to provide consent. Consent will comprise a dated signature from the woman and the dated signature of the person who obtained informed consent. A copy of the signed informed consent document will be given to the woman. A copy will be retained in the woman's medical notes, a copy retained by the Principal Investigator in the investigator site file (ISF). The process and timing for obtaining written consent will vary according to clinical urgency (see below)

An investigator will be available at all times to discuss concerns raised by women or clinicians during the course of the trial. Information about the trial will continue to be offered to women after they leave the hospital. A regular newsletter will be produced giving women, and their families, up to date information about the trial until it has finished.

4.1.1 Elective Caesarean Section

Eligible women requiring elective CS will be provided with further information and the opportunity to ask questions at the time the operation is booked and approached for written consent at pre-operative assessment clinic or on the day of surgery. It will be clearly stated that she is free to withdraw from the trial at any time, for any reason, without prejudice to future care and with no obligation to give the reason for withdrawal. Randomisation will take place on the day of surgery.

4.1.2 Emergency Caesarean Section

A substantial challenge to the conduct of an individual RCT of IOCS is obtaining consent from women in labour who require emergency CS. This population may be the very individuals who derive specific benefit from this health technology and therefore their participation is vital.

As detailed above, as many women as possible booked for delivery at participating centres will have received the information regarding the trial during their pregnancy so that sufficient time is given to consider participation in the trial should an emergency caesarean section be required. On admission to delivery suite, women's notes will be checked to ensure this information was supplied, and the opportunity for further discussion provided. Eligible women interested in taking part will have this indicated on their medical notes if this has not been done previously.

Despite best attempts to ensure the maximum number of women receive information during their pregnancy, some women may arrive on delivery suite who have not previously received trial information. As discussed in Section 1.3.7, many of these women perceive cell salvage as reassuring and potentially beneficial and will wish to enter the trial to have the opportunity of receiving it in the event that they require a caesarean section. If they are not distressed, these women may have prolonged periods during which they are capable of reading and considering the SALVO information sheet, and subsequently giving informed consent for recruitment; this includes women in early labour, or who have effective epidural analgesia in progress.

Approaching Women In Early Labour

In routine practice, women who attend the delivery suite for assessment and are found to be in the latent first stage of labour (early labour) are offered individualised support and occasionally analgesia, and encouraged to return home as per NICE guidelines (NICE CG55 intrapartum care, 2007) or sometimes they remain in the hospital. Under either of these circumstances, a woman who meets the following criteria (ie she is not in established labour) may be offered trial information by the midwife caring for her:

- Woman is willing to receive the trial information and is subsequently willing to discuss the PIS and have any questions answered if desired.

- 0-3cm dilation, not contracting regularly (ie a maximum of one contraction in ten minutes, with contraction lasting less than 30 seconds).
- She will be advised that the person taking informed consent will return after 1 hour to see if she would be interested in taking part, should she require a caesarean section. If her situation changes and labour becomes established during that hour, or if she requires a caesarean section before the hour has elapsed, she will not be approached for inclusion. (If the woman has a contraction during the discussion about the study, the clinician involved will pause and wait for the contraction to finish. Permission to continue with the discussion will then be sought.)
- After the discussion, it will be recorded whether the woman is or is not willing to take part in the trial, should a caesarean section be required.
- **Women in established labour (ie 4cm and regular painful contractions) will not be approached for the first time on delivery suite.**
- **Women who are distressed and not in a position to absorb the information on the patient leaflet will not be approached for the first time on delivery suite.**

Approaching women with epidural
If a woman in labour has effective epidural analgesia in progress and is comfortable, she may be offered trial information by the midwife caring for her if the following criteria are met:

- Woman is willing to receive the trial information and is subsequently willing to discuss the PIS and have any questions answered if desired.
- She will be advised that the person taking informed consent will return after 1 hour to see if she would be interested in taking part, should she require a caesarean section. If her situation changes and she has not remained comfortable for that hour, or if she requires a caesarean section before the hour has elapsed, she will not be approached for inclusion.
- After the discussion, it will be recorded whether the woman is or is not willing to take part in the trial, should a caesarean section be required.

Consent will be obtained if a decision for caesarean section is made (see below). It will be clearly stated that she is free to withdraw from the trial at any time, for any reason, without prejudice to future care and with no obligation to give the reason for withdrawal.

The majority of emergency CSs in the absence of acute fetal distress are conducted in a controlled manner with ample time for regional anaesthesia to be established. In this context, it is feasible for written consent to be obtained at this stage once the decision for CS has been made.

However, in some emergency situations, the urgency of the situation means that for some women, it may not be possible to obtain written consent for the trial prior to the emergency CS. In this instance, where there is insufficient time for written consent to be obtained but the woman has capacity to consent and has previously indicated an interest in taking part in the trial, verbal consent will be obtained. Written consent will then be completed once the urgency of the situation is over and the caesarean section complete.

Figure 5: Procedures Flow Chart

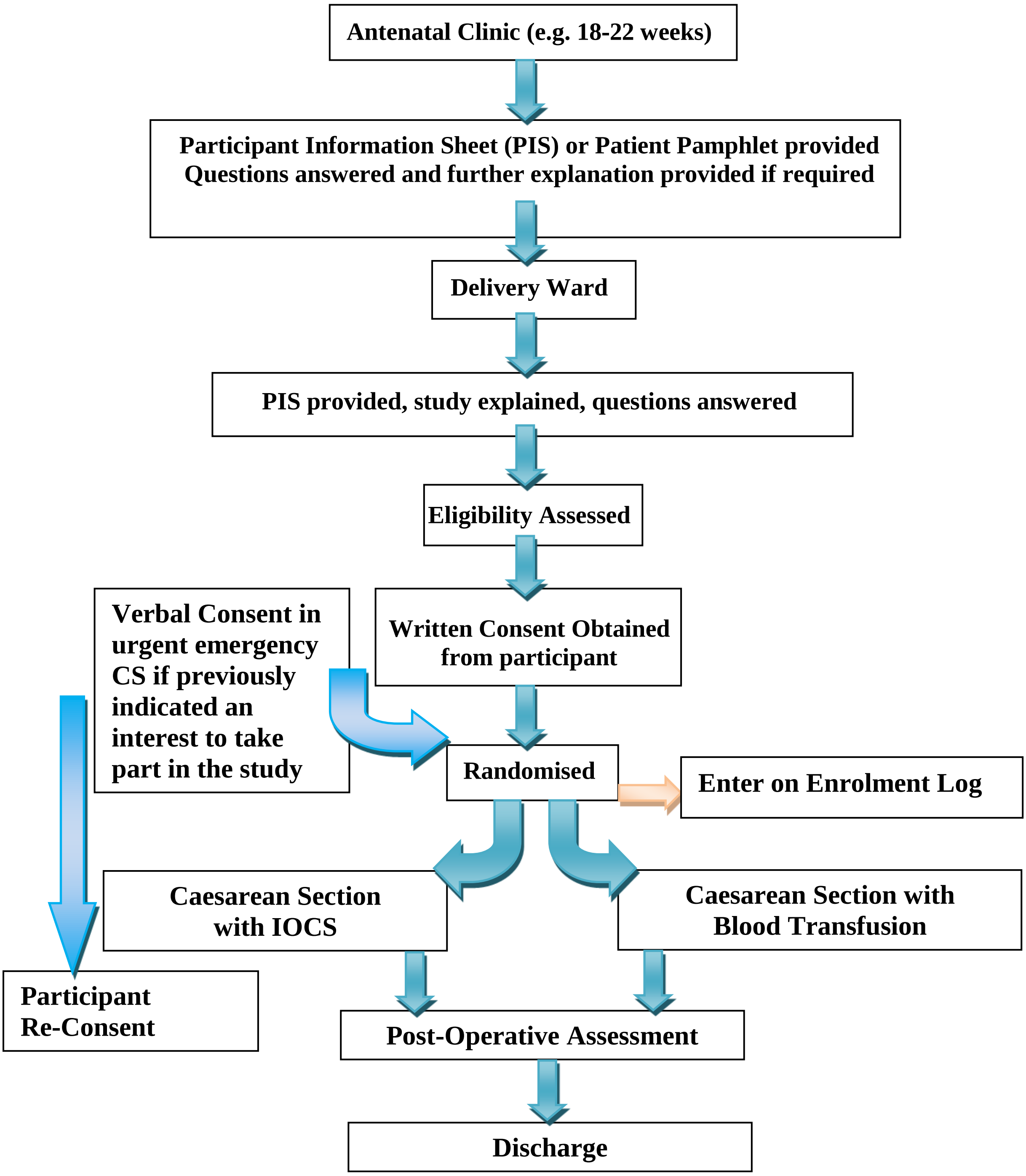

4.2 Screening Procedures

To confirm eligibility for randomisation, investigators will need to verify women meet the inclusion/exclusion criteria for the trial as well as gaining informed consent. An eligibility checklist will be completed on the Randomisation Form, prior to randomisation. Participants who provide informed consent and are eligible for randomisation will have key parameters such as gestational age, indication for caesarean section, obstetric history, demographics and prognostic factors collected.

4.3 Randomisation Procedures

Once eligibility has been confirmed and consent has been obtained, the participant can be randomised to either caesarean section with IOCS or caesarean section with standard care. Randomisation will occur on the delivery ward, at the time women are being prepared for theatre.

Randomisation to the allocated intervention (allocation ratio 1:1) will use a bespoke web-based randomisation system hosted by Bristol Randomised Trial Collaboration (BRTC), University of Bristol as detailed in the relevant trial standard operating procedure (SOP). The randomisation will use random permuted blocks of variable sizes to ensure that trial staff conducting randomisation cannot reliably predict the next allocation.

Randomisation will be stratified, by four criteria:
1. Centre
2. Type of Caesarean (Emergency/Elective)
3. Presence of abnormal placentation
4. Multiple births (twins or more)

The procedures for randomisation will be fully documented, tested and validated prior to the start of the trial and monitored by the BRTC during the trial. Once the participant has been successfully randomised to the study, they will be added to the enrolment log.

Each participating centre will have access to the randomisation system with a unique hospital pin number. This will be restricted to staff as delegated by the Principal Investigator. They will input the details required for randomisation and immediate on-screen randomisation will occur. An email will be automatically generated to notify the chief investigator when each participant is randomised to the trial. This will be a blinded email devoid of any allocation information.

4.4 Methods for minimising the risk of bias

Allocation concealment with third party randomisation will help minimise *selection bias*.

Performance bias may lead transfusion rates to vary. We will minimise this risk by ensuring that each centre has an intra-operative transfusion protocol for use in theatre and recovery to standardise operative transfusion triggers across both study groups in each centre. A similar standardised checklist will be used to guide decisions on post-operative transfusion (See appendix 3 for example). Some centres may adopt an agreed haemoglobin threshold for transfusion, which should be applied equally to both groups. There is evidence that the presence of symptoms such as lassitude, dyspnoea and fatigue provide excellent surrogates to haemoglobin estimation to guide donor blood use (24). Maternal activity in the period immediately after CS and the demands of the tasks associated with care of the new-born are unlike most other contexts of recovery from an operative intervention where greater inactivity is considered the norm. To be pragmatic, the protocol may be allowed to vary between centres according to consensus opinion amongst clinicians. This represents real world variation in local practices and will not contribute to bias.

An attempt will be made to blind post natal carers to group allocation after CS. The allocation will not be recorded in routine case notes but this does not represent formal blinding as theatre notes will be available. The carers on post natal wards are a different group of staff to the carers on labour wards and operating theatres. It is on the post natal wards where the decisions for post-operative donor blood transfusions are made. This is based on the post-operative haemoglobin level and maternal symptoms. In the event of the need for a donor blood transfusion, serum haemoglobin (Hb) will be measured by blood sample, pre and post-transfusion and results recorded. This will allow monitoring of numeric transfusion thresholds between units and groups. In the unlikely event that between group variations in haemoglobin transfusion triggers were indeed evident, consideration would be given for adjusting for such differences in the final analysis. It is worthy of note that alternative trial designs, such as cluster randomisation, would not necessarily minimise the performance bias problem (section 2.2 on choice of design); it would simply reduce the power.

4.5 Preparing and supporting theatre and medical staff for participation in the trial

The use of the IOCS machine represents an additional task for theatre personnel and medical staff to undertake during a caesarean section, which is of particular importance in an emergency situation. It will therefore be crucial to the success of the trial that all staff are sufficiently trained and familiar in the use of the IOCS machine. IOCS technology is already present at participating centres with many theatre staff skilled in its use. Training will be conducted by manufacturers of the IOCS machine and the trial coordinating centre prior to recruitment of participants, at site initiation and training visits.

4.6 Schedule of Treatment

Women will be randomly allocated to either:

1. Caesarean section with IOCS (intervention group), set up routinely with collection of shed blood from the outset of surgery, and return of any processed blood obtained.
2. Caesarean section without IOCS (control group), with transfusion of donor blood according to standard local guidelines.

Blood will be aspirated from the surgical field; the red cell component isolated by centrifugation and after washing and filtration, re-transfused. The ability to return salvaged blood is dependent on sufficient volume being collected and processed. Blood will be uniformly returned to women in the IOCS group if this volume threshold is reached. The control group will receive standard current practice (without IOCS), which may involve donor blood transfusion. In life threatening acute haemorrhage, women will be managed at the discretion of attending clinicians, in line with Centre for Maternal and Child Enquiries (CEMACE) guidance, potentially including the use of IOCS in the control arm. We anticipate this to be a very rare occurrence. The indications for postoperative donor blood transfusion will be set according to local protocols in each hospital and deviations from this criterion will be monitored.

4.7 Table 3: Schedule of Assessment

	**Pre-operative**	**Intra- operative**	**Post-operative (24 hours)**	**Discharge**
**SCREENING**				
Eligibility	X			
Informed consent	X			
Obstetric History/Prognostic Factors/<br>Indication CS/Demographics	X<br>X			
Randomisation	X			
**TRANSFUSION**				
Transfusion of ≥1 unit donor blood		X	X	
Number of donor blood units transfused		X	X	
Volume of blood returned by IOCS		X		
IOCS consumables used		X		
IOCS technical failure		X		
**MATERNAL**				
Haemoglobin level	X		X	
Maternal exposure to fetal blood (Kleihauer test)			X	
Transfusion reaction			X	
Anti D requirement and dose			X	
Time to first mobilisation after CS			X	
Multidimensional Fatigue Inventory (MFI)				X
**HEALTHCARE UTILISATION**				
Length of hospital stay				X
Adverse Events		X	X	X

4.8 Criteria for Early Termination of the study

The short-term nature of the intervention and follow-up makes it unlikely that any new information will be of relevance to an individual participant. As far as we are aware, there are no similar studies recruiting obstetric participants. In the unlikely event that the cell salvage machine manufacturers (with whom we have good links) issue an important safety notice, we will suspend recruitment to the trial. Additionally, if the DMC committee, TSC, REC or sponsor determine it is within the best interests of the participants or trial to terminate the study, written notification will be given to the CI. This may be due to, but not limited to; safety concerns, proof of efficacy or non-compliance/serious breaches. If the study is terminated participants will be returned to the NHS normal follow up and routine care for IOCS and CS procedures.

4.9 End of Study Definition

When the last enrolled participant has been discharged from hospital, the REC will be notified of the trial completion. The final study report will be completed 6 months after the trial completion.

5 Laboratories (if applicable)

5.1 Central/Local Laboratories

NHS pathology services will conduct all study laboratory analysis.

5.2 Sample Collection/Labelling/Logging

Sample collection requirements include full blood count (FBC) for analysis of haemoglobin levels at both pre and post-operative time points. A Kleihauer test is also required to measure maternal exposure to fetal blood postoperatively. The requirement for anti-D will be determined by analysis of maternal and fetal blood types and rhesus status postoperatively. These tests are considered to be standard care for women undergoing caesarean section and do not constitute additional tests for trial purposes. Samples will be collected, labelled and processed according to NHS standard practice and logged onto the NHS database.

5.3 Sample Receipt/Chain of Custody/Accountability

Samples will be checked by laboratory staff as per NHS standard practice prior to processing. Any inconsistencies will be referred back to the person collecting the sample or the research team. All samples received and processed will be logged onto the NHS database.

5.4 Sample Analysis Procedures

Sample analysis will be conducted according to the NHS standard operating procedures.

5.5 Sample Storage Procedures (if applicable)

All samples will be processed upon receipt. No sample storage is required.

5.6 Data Recording/Reporting

Pathology reports will be printed and filed in the participants medical records as per usual NHS practice. The haemoglobin and Kleihauer results will then be transcribed to the trial CRF by a delegated member of the trial team. The CRF will be pseudonymised with all participant identifiers removed.

6 Pharmacovigilance

6.1 General Definitions

6.1.1 Adverse Event (AE)

An AE is any untoward medical occurrence in a participant receiving trial intervention, including occurrences which are not necessarily caused by or related to that intervention. An AE can therefore be any unfavourable and unintended sign (including an abnormal laboratory finding), symptom or disease temporarily associated with study activities.

6.1.2 Serious Adverse Event (SAE)

An SAE fulfils at least one of the following criteria in the context of the SALVO trial:

- Is fatal – results in death (NOTE: death is an outcome, not an event)
- Is life-threatening
- Requires prolongation of hospitalisation after CS beyond 7 nights.
- Results in persistent or significant disability/incapacity
- Is a congenital anomaly/birth defect
- Is otherwise considered medically significant by the Investigator

6.2 Investigators Assessment

6.2.1 Seriousness

The local Principal Investigator is responsible for the care of the participant, or in his absence an authorised medic within the research team, is responsible for assessing whether the event is serious according to the definitions given in section 6.1.

6.2.2 Causality

The Investigator must assess the causality of all serious adverse events in relation to the trial treatment according to the definition given

6.2.3 Expectedness

The investigator must assess the expectedness of all SAEs according to the definition given. If the SAE is unexpected and related, then it needs immediate reporting.

6.2.4 Severity

The Investigator must assess the severity of the event according to the following terms and assessments. The intensity of an event should not be confused with the term “serious” which is a regulatory definition based on patient/event outcome criteria.

Mild: Some discomfort noted but without disruption of daily life
Moderate: Discomfort enough to affect/reduce normal activity

Severe: Complete inability to perform daily activities and lead a normal life

6.3 Notification and reporting Adverse Events or Reactions

If the AE is not defined as SERIOUS, the AE is documented in the participants' medical notes (where appropriate) and the trial AE log and the participant is followed up by the research team.

6.4 Notification and Reporting of Serious Adverse Events

All SAEs occurring during the trial observed by the investigator or reported by the participant, whether or not attributed to the trial, will be documented in the participants' medical notes (where appropriate) and reported on the AE log for the trial. All SAEs will be followed up until resolution or the event is considered stable. The investigator may be asked to provide follow-up information. All related SAEs that result in a participant's withdrawal from the trial or are present at the end of the trial, should be followed up until a satisfactory resolution occurs.

A maximum hospital stay following CS is considered to be 7 days. Any stay longer than 7 days should be considered prolongation of existing hospitalisation and recorded as an SAE on the AE log.

Local Principal Investigators are responsible for reporting SAEs to the chief investigator and their host institution, according to local regulations. All SAE's are to be reported to the CI within 24 hours of learning of the event. The CI must then report SAE's that are considered to be 'related' and 'unexpected' to the PCTU QA manager and to the sponsor within the 24 hours and then to the Main REC within 15 days in line with the required timeframe and sponsor and PCTU SOPs. For further guidance on this matter, please refer to Appendix 1.

6.4.1 Expected Adverse Events

Although no serious adverse events are anticipated, it is possible that these may occur.

Risks related to trial procedures include;

- Maternal exposure to fetal blood,
- Amniotic fluid embolism and
- Severe hypotension
- Transfusion reaction,

These are considered low level risks that are unlikely to occur with increased frequency for trial participants. Should they occur with severity meeting the criteria for a serious adverse event, they will be reported as a related and unexpected SAE.

6.5 Urgent Safety Measures

The CI may take urgent safety measures to ensure the safety and protection of the clinical trial participants from any immediate hazard to their health and safety, in accordance with Regulation 30. The measures should be taken immediately. In this instance, the approval of the Licensing Authority Approval prior to implementing these safety measures is not required. However, it is the responsibility of the CI to inform the sponsor and MREC (via telephone) of this event immediately.

The CI has an obligation to inform the MREC in writing within 3 days, in the form of a substantial amendment. The sponsor (JRO) and PCTU QA manager must be sent a copy of the correspondence with regards to this matter. For further guidance on this matter, please refer to Appendix 2

6.6 Annual Safety Reporting

The CI will send the Annual Progress Report to the MREC using the NRES template (the anniversary date is the date on the MREC "favourable opinion" letter from the MREC) and to the sponsor. Please see Appendix 2 for further information. The CI will report a cumulative line listing of all related and unexpected SAEs to the MREC annually, and to the DMC and Trial Steering Committee every 6-12 months. The DMC will view data with knowledge of treatment. If a participant dies as a result of the study protocol or study interventions, any post-mortem findings must be provided to the Chief Investigator, who will report the findings to the DMC for continuous safety review.

6.7 Overview of the Safety Reporting Process/Pharmacovigilance responsibilities

The CI has the overall pharmacovigilance oversight responsibility. The CI has a duty to ensure that pharmacovigilance monitoring and reporting is conducted in accordance with the sponsor's requirements and the PCTU SOP. An organogram of the communication of SAEs is shown in Appendix 1. Each participating trial site will be responsible for reporting all SAEs to the chief investigator immediately so that a decision can be made as to whether this needs to be reported to the sponsor, PCTU QA manager and MREC. The CI will keep a log of all SAE's reported by the participating centres for reporting to the REC and DMC.

7 Statistical Considerations

7.1 Trial Outcomes

7.1.1 Primary outcome

The primary outcome is the proportion of women needing donor blood transfusion to deal with haemorrhage and its consequences. (see section 3.5).

7.1.2 Secondary outcomes

- Severity of events quantified as the volume of blood transfused
- Time to first mobilisation after CS
- Length of hospital stay
- Multidimensional Fatigue Inventory (MFI)
- Resources used intra- and post-operatively, including IOCS consumables and donor blood transfusions
- Costs of staff training, service procurement and provision of care will be collected alongside clinical outcomes

7.1.3 Safety outcomes

- Pre- and post-operative serum haemoglobin, and mean fall in haemoglobin level
- Maternal exposure to fetal blood measured by Kleihauer test
- Requirement for and the dose of anti-D antibody administered

7.1.4 Process outcomes

- Volume of blood returned in IOCS (mean/SD)
- Proportion of transfusion reaction associated with allogeneic donor blood transfusion
- Episodes of technical failure of IOCS

7.2 Primary Analysis

A detailed analysis plan will be developed and agreed by the Trial Steering Committee and the Data Monitoring Committee, prior to unblinding and data analysis. The primary analysis will be a comparison of the management policies assigned at randomisation (intention-to-treat). The risk of the primary outcome in the IOCS group will be compared with the usual practice group and tested for significance at the two-sided 5% level of significance. Crude and adjusted odds ratios and 95% confidence intervals will be produced (The adjusted analysis will be considered the primary analysis).

The analysis will be adjusted, to allow for known or suspected prognostic factors, to be specified in the analysis plan. Results will be reported according to the Gantt chart, Section 9.4.1.

The following subgroup analyses have been pre-specified for the primary outcome:

- analysis of treatment effect by indication for Caesarean Section.
- analysis of treatment effect by recruitment Centre.

The consistency of the treatment effect across subgroups will be explored using the statistical test of interaction. Further exploratory analyses will also be undertaken after the main trial report is complete. These will include an exploration of whether there are specific prognostic factors for intervention benefit or adverse outcomes, using tests of interaction. These analyses will be hypothesis-generating and the findings will be interpreted cautiously.

As a sensitivity analysis, we will analyse the primary outcome assuming that all instances of the use of IOCS in the control arm would have been instances of donor blood transfusion had the IOCS machine not been present.

7.3 Secondary Analysis

Analysis of secondary outcomes will be clearly delineated from the primary analysis in any statistical reports produced. Estimates of treatment effect (unadjusted, and adjusted for the same covariates as in the primary analysis) will be obtained using methods appropriate to the scale of measurement of the outcome (see Section 7.5). Analyses will be intention-to-treat.

7.4 Safety Endpoints

Adverse events will be recorded from the time of randomisation to the time of discharge. Safety outcomes will be compared between the groups, as for secondary endpoints.

7.5 Sample Size

The proposed sample size is a total of 3,050 women (1,525 per group). To detect an absolute difference of 2% in the transfusion rate as defined in Section 7.1.1 (5% in the standard care group, 3% in the IOCS group, relative risk 0.6) this sample size would give 80% power for a 2-sided test (Figure 6). In reaching this estimate we have considered definition of primary outcome, control event rate, expected effect size, choice of study design, power and directionality of hypothesis.

Figure 6: Power Curves

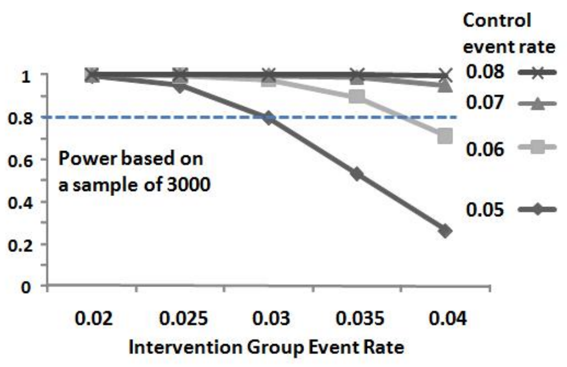

Establishing a baseline rate for the primary outcome is not straight forward since estimates in the published literature for blood transfusion in CS vary widely (1.8% to 23.5%) (2;3). Factors influencing this figure include country of origin, indication for CS and local transfusion policy. Contemporary observational reports put transfusion rates for an unselected CS population at around 5% in current UK practice. Our audit in two investigating centres has shown around a 5% transfusion rate. Our pilot sample is too small to assist in providing reliable information on sample size calculations. In the light of reported contemporary observations and audited data on transfusion rates, the assumption of a 5% event rate has been used to base the main sample size recalculation on.

The expected effect estimate can be derived from the literature. Our systematic review (1) and its most recent update has shown only one small trial published in 1998 (3), which randomised a total of 68 participants to either IOCS or standard care. The transfusion rate in the control group was 23.5% and 2.9% in the IOCS group. The control event rate is considerably higher than that observed in current UK practice and inconsistent with literature from other sources. It is likely to be due to a sample at exceptionally high risk of haemorrhage. Weaknesses that raise the risk of bias (e.g. inadequate concealment of randomisation) preclude reliance on it alone to inform our calculations. Non-obstetric literature evaluating IOCS in interventions with a moderate to high risk of transfusion has two high quality systematic reviews: a HTA report citing a RR of exposure to allogeneic blood of 0.59 (95% CI 0.48-0.73) with salvage (6); and a Cochrane review reporting a RR of 0.62 (95% CI 0.50-0.70) for transfusion with salvage compared with normal practice (7). Detecting smaller effect size is possible but the larger sample size required has to be balanced against the cost and practicability of undertaking such a trial. From the current best literature we assume an intervention effect at or around 0.6 (at a control event rate of 5%, the intervention group would have a transfusion rate of 3%).

We have discussed the issue of directionality of hypothesis and significance testing and provided a range of sample sizes in table 6. Figure 6 shows how the power of our 3050 sample changes across a range of control event rates (between 4-7%), experimental event rates (between 2-4%) and directionality of hypothesis (1- or 2-sided test).

Table 6 Sample size estimates using a range of control event rates and an RR of 0.6 for transfusion at 80% and 90% power based on one and two sided tests

Control event rate	Two sided test Power 80%	Two sided test Power 90%	One sided test Power 80%	One sided test Power 90%
7%	2116	2830	1666	2308
6%	2490	3332	1960	2714
5%	3012	4032	2374	3286
3%	5106	6836	4022	5570

The interim analysis of our pilot study has confirmed that primary outcome data can be obtained in virtually all the randomised women, since this information is collected at the end of the in-patient stay before hospital discharge. Our sample size allows for primary outcome data and follow up loss of around 1% (38) randomised cases.

7.6 Statistical Analysis

A detailed analysis plan will be developed and agreed by the Trial Steering Committee and the Data Monitoring Committee, prior to unblinding and data analysis.

Demographic factors and clinical characteristics will be summarised with counts (percentages) for categorical variables, mean (standard deviation[SD]) for normally distributed continuous variables, or median (interquartile [IQR] or entire range) for other continuous variables. Numbers of participants who are eligible, recruited, and followed up will be recorded in a CONSORT flow-chart.

Adjusted and unadjusted (crude) treatment effects will be estimated using multivariable and univariate regression analysis, respectively. Binary outcomes (peripartum transfusion, requirement for anti-D antibody and any other binary secondary outcomes) will be analysed with logistic regression. Quantitative outcomes where a symmetric, unimodal distribution is expected (number of units transfused, volume of blood transfused, serum haemoglobin, Multidimensional Fatigue Inventory will be analysed with linear regression. Quantitative outcomes with strongly skewed distributions (maternal exposure to fetal blood, dose of anti-D antibody) will have cut-offs defined in the analysis plan and will be analysed as binary variables. Time-to-event outcomes (time to first mobilisation, length of hospital stay) will be analysed with Cox proportional hazards regression. The analysis of post-operative serum haemoglobin will allow for change from baseline by including the pre-operative level as an additional covariate. Differences between treatment effects in different subgroups (in planned subgroup analyses and in exploratory analyses) will be analysed with tests for interactions. $P<0.05$ will be used to determine statistical significance in all analyses.

Analyses will be intention-to-treat; for this reason every effort will be made to collect complete data for all randomised participants. Where baseline covariates are missing we will use mean imputation of the covariate or a missing indicator covariate in adjusted analyses (note that epidemiological arguments against the use of a missing indicator do not apply in randomised trials) (27) An intention-to-treat approach does not mean that all outcome data must have been collected (28), though pilot work for this trial suggests that all or close to all of the primary outcome data will be obtained. Where outcome data are missing we will analyze those who do have outcome data, adjusting for baseline covariates. This approach is unbiased if missingness for the outcome is related to observed covariates

("missing at random"). Further sensitivity analyses will be used if necessary to explore the missing at random assumption.

The study statistician and chief investigator will remain blinded so as not to bias the analysis and interpretation of results. An independent statistician employed by the PCTU will provide the DMC statistician with the key to unblind the data, and the study statistician will provide computer code to allow the DMC statistician to produce unblinded summaries as required by the DMC.

7.7 Health Economics Analysis

The aim of the economic evaluation is to determine the relative cost-effectiveness of the IOCS compared to current practice. The economic evaluation will be carried out from the perspective of the NHS therefore only direct costs and outcomes to the health service associated with the intervention will be included in the analysis and these direct costs will include those associated with adverse events. The evaluation will consider costs incurred by the health service in the delivery of the alternative treatment pathways and given the duration of follow up in the trial is for the immediate post natal period until discharge from hospital, it is a pragmatic decision to limit the perspective to the NHS. There is no reason to expect the alternative strategies to cause significant variation in the private costs to individuals, or to society, between the arms of the study beyond this time point, so wider costs such as primary data on the private out of pocket costs to individuals associated with the study will not be collected.

7.7.1 Cost data collection

Data collection will be undertaken prospectively at all units participating in the trial. The process of collecting resource use data will be undertaken separately from data collection on unit costs.
The main resource use to be monitored includes the following:

1) The duration of health service staff time spent carrying out IOCS procedure including staff training
2) Equipment and disposables required for the IOCS procedure.
3) Number of donor blood transfused units required by participants,
4) Length and type of hospital inpatient stay (Any adverse events will increase the length and type of hospital stay)

Unit costs will be obtained and attached to resource items in order that a cost can be calculated for each caesarean section. Unit costs will be obtained from published sources and centres participating in the trial. Published sources will include Unit Costs of Health and Social Care (30) and NHS Reference cost. Costs used in other relevant published sources will be sought for use in sensitivity analysis.

7.7.2 Analysis

Given the objective of the trial and the duration of follow up, only a within trial economic analysis will be carried out. A preliminary cost consequence analysis will compare all costs and outcomes for the intervention and current practice in a disaggregated format. The main economic analysis will be in the form of a cost effectiveness analysis based on the outcome of cost per donor blood transfusion avoided.

The analysis will adopt an incremental approach in that data collection will concentrate on resource use and outcome differences between trial arms. As the majority of cost data are skewed, and the mean cost of each procedure is of importance, a bootstrapping approach will be undertaken in order to calculate confidence intervals around the mean costs. The recommended approach to discounting will be followed if necessary, which would include discounting costs and benefits as per NICE guidelines at 3.5%. But as the trial and analysis are limited to the immediate post natal period, and therefore not likely to extend beyond 12 months, this process in not likely to be necessary.

If appropriate, we will present results of all economic analyses using cost-effectiveness acceptability curves to reflect sampling variation and uncertainties in the appropriate threshold cost-effectiveness value where appropriate. Uncertainty in the confidence to be placed on the results of the economic analysis will be explored by estimating. These plot the probability that the intervention is cost effective against threshold values for cost effectiveness. The robustness of the results will be explored using sensitivity analysis. This will explore uncertainties in the trial based data itself, the methods employed to analyse the data and the generalisability of the results to other settings.

8 Data Handling & Record Keeping

8.1 Confidentiality

The Chief Investigator is the 'Custodian' of the data and will ensure that information with regards to study participants will be kept confidential and managed in accordance with the Data Protection Act, NHS Caldicott Guardian, The Research Governance Framework for Health and Social Care and Research Ethics Committee Approval.

Identifiable information to be collected from the participants include, full name, DOB and hospital number and contact details at screening. This information will be used to contact participants but will not leave the study site. All case report forms will be pseudonymised.

The trial data will be made available to suitably qualified members of the research team, study monitors and auditors, the REC and regulatory authorities as far as required by law.

The participants will be anonymised with regards to any future publications relating to this study.

8.2 Required Study Documents

- A signed protocol and any subsequent amendments
- PCTU self-monitoring template for the trial team to complete on a regular basis as detailed by the Trial Monitoring section
- Current/Superseded Patient Information Sheets (as applicable)
- Current/Superseded Consent Forms (as applicable)
- Indemnity documentation from sponsor
- Conditions of Sponsorship from sponsor
- Conditional/Final R&D Approval
- Signed site agreements
- Ethics submissions/approvals/correspondence
- CVs of CI and site staff
- Laboratory accreditation letter, certification and normal ranges for all laboratories to be utilised in the study
- Delegation log
- Staff training log
- Site signature log
- Identification log
- Enrolment log
- Monitoring visit log
- Protocol training log
- Correspondence relating to the trial
- Communication Plan between the CI/PI and members of the study team

- SAE reporting plan for the study

8.3 Case Report Forms

For all participating women, pre-operative trial data will be recorded in the maternity notes which will be completed by the attending obstetrician, anaesthetist or midwife. The following data will be abstracted from the notes and recorded on CRF for the trial, relying on CLRN research support in each of the recruiting hospitals.

Pre-Operative

- Demographic data
- Obstetric history
- Gestational Age/Prognostic factors/Indication for CS
- Pre-op Hb level

Intra/Post-Operative

- AE/SAE Log

Further information will be collected during intra-operative and post-operative phase and at the time of discharge from hospital. This data will be recorded directly to trial CRF's by the attending obstetrician, anaesthetist or midwife as outlined on the trial delegation log. Any missing information will be obtained from trial participant prior to discharge. Checking of this data, along with administration of multidimensional fatigue inventory on the post natal wards, will rely on CLRN research support in each of the recruiting hospitals. No further routine follow up will be required after discharge.

The following trial specific data which will be entered directly to CRF:

Pre-Operative

- Eligibility criteria checklist

Intra-Operative

- Transfusion of ≥1 unit donor blood
- Number of donor blood units transfused
- Volume of blood returned by IOCS
- IOCS consumables used
- IOCS technical failure
- Maternal exposure to fetal blood

Post-Operative

- Transfusion of ≥1 unit donor blood
- Number of donor blood units transfused
- Hb level
- Transfusion reaction
- Anti D requirement and dose
- Time to first mobilisation after CS

Discharge

- Multidimensional Fatigue Inventory (MFI)
- Length of stay in Hospital

Other

- Additional Information/Note to File CRF
- Final Study Status /Early Withdrawal CRF

Suitably qualified members of the study team, as documented on the trial delegation log will be responsible for the completion of the CRFs. CRFs will be pseudonymised using a participant code allocated at time of randomisation. The number will be generated by the online randomisation system and recorded on the Randomisation Form. This code will consist of the trial site code followed by the consecutive recruitment number starting at 001. Site codes are documented in Appendix 4.

E.g.: Royal London Hospital (01), participant number 1 (001) : 01001

8.4 Data collection, processing and monitoring

All trial data will be managed according to the PCTU data management SOP's.
Data will be;

- Collected using case report forms as outlined in section 8.3.
- Verified and processed on site by trial coordinators or other delegated members of the study team for data entry to the trial database.
- Monitored centrally for consistency, viability and quality by the PCTU.
- Screened for out-of-range data, with cross-checks for conflicting data within and between CRF using computerised logic checking screens
- Referred back to the relevant centre for clarification in the event of missing items or uncertainty

8.5 Central statistical monitoring

All data will be monitored centrally (at the PCTU) for consistency, viability and quality using bespoke data management systems. Central statistical monitoring will examine patterns of recruitment at sites, characteristics of women, time of recruitment, etc. The trial programmer will run trial-specific programs to extract certain fields from the database (as requested by the Chief Investigator or Trial Statistician) and to cross-check specific information. These fields may include measures of eligibility criteria, management after trial entry and compliance but not by allocation. The trial programmer and Chief Investigator will review the results generated for logic and for any patterns or problems. Outlier data will be investigated. The Chief Investigator and Trial Statistician will decide if any action is required

8.6 Record Retention and Archiving

During the course of research, all records are the responsibility of the Chief Investigator and must be kept in secure conditions. When the trial is complete, it is a requirement of the Research Governance Framework and Trust Policy that the records are kept for a further 20 years. For trials involving BLT Trust patients, undertaken by Trust staff, or sponsored by BLT or QMUL, the approved repository for long-term storage of local records is the Trust Modern Records Centre which is based at 9 Prescot Street. Site files from other sites must be archived at that external site and cannot be stored at the Modern Records Centre.

8.7 Quality Control and Quality Assurance

8.7.1 Summary Monitoring Plan

The study sites will perform remote trial monitoring according to the agreed PCTU trial monitoring plan and self-monitoring template. The frequency and intensity will be determined by the PCTU monitoring plan and risk assessment. Trial monitoring will include source data verification checks on informed consent forms and eligibility for randomisation and a sample set of CRFs. The remote

monitoring reports reviewed by the PCTU and all findings will be followed up and actioned as per the trial monitoring plan.

The study sites will return self-monitoring templates to the PCTU every six months. The PCTU will also carry out triggered audits as determined by risk assessment or through findings identified via the remote monitoring reports. A random sample of cases will be monitored at source when site visits are performed. The documents to be verified will be randomly selected. Any major discrepancies found at a site visit would trigger a more extensive audit of trial data at the site involved. In addition, the sponsor may also carry out an audit throughout the duration of the trial.

8.7.2 Audit and Inspection

Auditing: Definition “A systematic and independent examination of trial related activities and documents to determine whether the evaluated trial related activities were conducted, and the data were recorded, analysed and accurately reported according to the protocol, sponsor's standard operating procedures (SOPs), Good Clinical Practice (GCP), and the applicable regulatory requirement(s).”

A study may be identified for audit by any method listed below:
1. A project may be identified via the risk assessment process.
2. An individual investigator or department may request an audit.
3. A project may be identified via an allegation of research misconduct or fraud or a suspected breach of regulations.
4. Projects may be selected at random. The Department of Health states that Trusts should be auditing a minimum of 10% of all research projects.
5. Projects may be randomly selected for audit by an external organisation.

Internal audits will be conducted by a sponsor’s representative

8.7.3 Compliance

The CI will ensure that the trial is conducted in compliance with the principles of the Declaration of Helsinki (1996), and in accordance with all applicable regulatory requirements including but not limited to the Research Governance Framework, GCP, Trust and Research Office policies and procedures and any subsequent amendments.

8.7.4 Non-Compliance

Definition - A noted systematic lack of both the CI and the study staff adhering to the principles of the Declaration of Helsinki (1996), applicable regulatory requirements including but not limited to the Research Governance Framework, GCP, Trust and Research Office policies and procedures and any subsequent amendments, which leads to prolonged collection of deviations, breaches or suspected fraud.

These non-compliances may be captured from a variety of different sources including monitoring visits, CRFs, communications and updates. The sponsor will maintain a log of the non-compliances to ascertain if there are any trends developing or escalating. The sponsor will assess the non-compliances and action a timeframe in which they need to be dealt with. Each action will be given a different timeframe dependent on the severity. If the actions are not dealt with accordingly, the sponsor will agree an appropriate action, including an on-site audit.

9 Clinical Governance Issues

9.1.1 Ethical Considerations

This protocol and any subsequent amendments, along with any accompanying material provided to the participant in addition to any advertising material will be submitted by the Investigator to an Independent Research Ethics Committee. Written Approval from the Committee will be obtained and subsequently submitted to the JRO to obtain Final R&D approval. The trial can only start after approval from a Research Ethics Committee and the local R&D "Sign-off" from each of the participating centres. If there is any further safety information which may result in significant changes in the risk/benefit analysis, the PIS and Informed Consent Form (ICF) will be updated accordingly and submitted to REC for revision and approval. All participants that are actively enrolled on the study will be informed of the updated information and given a revised copy of the PIS/ICF in order to confirm their wish to continue on the study.

9.1.1 Network Collaboration

Each recruiting unit will liaise with its regional CLRN. The lead CLRN will be the Central and East London CLRN. We will also be able to make use of the networks RM&G resources. Staff working on portfolio registered trials are eligible for the training courses which the UKCRN offer. The support costs for portfolio studies will ensure liaison of clinical staff with the research staff to ensure smooth running throughout. This will be a high accrual, complex intervention trial which networks will be obliged to facilitate. Experience from the recent deployment of portfolio studies suggests that service support costs and resources for additional research personnel are concrete benefits which can be derived from CLRN. For example, Birmingham & Black Country and South Yorkshire CLRNs deploy directly employed core research nurses to support national portfolio trials and ensure recruitment. We have confirmed support for research enquiry in this area from several national bodies including the Royal College of Obstetricians and Gynaecologists, the Obstetric Anaesthetists Association, the UK Cell Salvage Action Group (UKSAG) and the National Blood Transfusion Service. The National Childbirth Trust has provided consumer representation.

9.1.2 National registration systems

All women recruited into the trial will be 'flagged' after discharge. Information held by NHS and records maintained by the NHS Information Centre and Central Register may be used to help contact participants and provide information about their health status. Participants will be informed of this at the time of informed consent and permission sought to be contacted in the future.

9.1.3 Funding and Financial Aspects of the Trial

The study will be funded by the National Institute for Health Research Health Technology Assessment Programme (HTA), ref: 10/57/32

10 Trial Committees

The trial will be run on a day-to-day basis by the Trial Management Group (TMG) and supported by the Pragmatic Clinical Trials Unit (PCTU). The TMG reports to the Trial Steering Committee (TSC) which is responsible to the trial sponsor. At each participating centre, local Principal Investigators will report to the TMG via the UKCLRN project funded trial coordinator/research nurse.

10.1 Clinical Investigators Group

The Clinical Investigators Group (CIG) comprises the co-applicants, the principal investigators (PIs) and clinical investigators from each study site, the study clinical consultants, the study health economist, the study co-ordinators, the study statistician, the senior research health professionals and

other project staff. The CIG will meet periodically (every 3-6 months) but more frequently during trial set-up.

10.2 Local Co-ordination

Each participating centre will identify a site specific PI who will nominate a local co-ordinator for that centre (this may be him/herself) whose responsibilities will be to:

- Be familiar with the Trial
- Liaise with the PCTU and TMG.
- Ensure that all staff involved in the care of eligible women are informed about the trial and have received requisite training
- Ensure that mechanisms for recruitment of eligible women, including the availability of patient information, are in place; monitor their effectiveness and discuss the reasons for non-recruitment with relevant staff
- Notify the CI of any SAE's or SUSAR's
- Make data available for verification, audit and inspection processes as necessary
- Ensure that the confidentiality of all information about trial participants is respected by all persons

10.3 Trial Management Group

The Trial Management Group (TMG) comprises the study chief investigator (CI), the trial manager, the study health economist, the study coordinators, the study statistician, the senior research healthcare professionals and other project staff. The TMG staff will hold regular meetings (every 3-4 weeks).

10.3.1 Site set-up and training

Start-up visits at each site, including training in trial procedures, will be performed before participants being enrolled at that site. Training will be documented on SOP training logs and protocol training logs. Regular site visits will be made by the members of the trial management group to ensure adherence to the protocol and to deal with any specific site issues. Staff trial training days will be undertaken to ensure that staff involved with the trial are fully appraised of issues such as consent, compliance with the protocol, data collection and changing regulations. An annual meeting for principal investigators and research staff will be organised with workshops to discuss protocol issues, data collection issues and how trial specific procedures are conducted.

10.3.2 Project timetable, milestones and projected recruitment

From recent experience in the PulseOx and BUMPES studies, we anticipate it will take 6 months to recruit research staff and obtain MREC and research governance approval for the sites involved. Central trial personnel will include a trial coordinator and clinical consultant, who will supervise local research personnel at each site. Local staff will be trained to provide information to expectant mothers, recruit women to the trial, train theatre staff in IOCS and data collection. The trial will be conducted in at least 17 large regional obstetric units. Units of this size perform at least 1500 Caesarean sections per year. A recruitment target of 3050 over two years would require each unit to contribute 90 women per year or about 8 patients a month (Figure 7).

Figure 7 : Accrual Projection

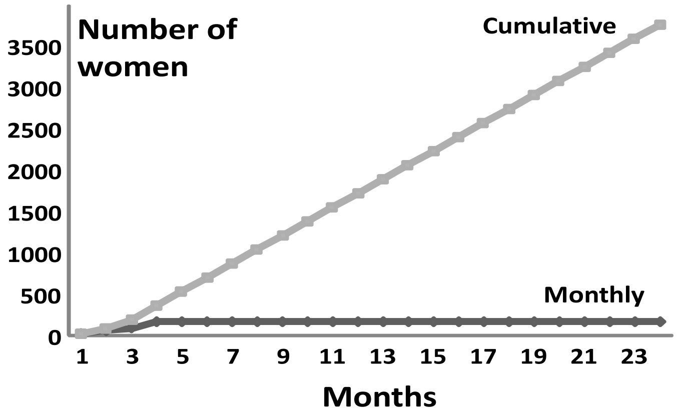

This conservative estimate is reasonable considering the challenges of recruiting women in labour. A data monitoring committee will report every 6 months to an independent trial steering committee. Data entry and analysis, economic evaluation, dissemination of research findings and report writing will take 6 months. The total length of the study will therefore be 3 years and cost £661 per woman recruited (Table 7)

Table 7: Gantt Chart of Project Milestones

Project Years	Year 1												Year 2												Year 3											
**Months**	1	2	3	4	5	6	7	8	9	10	11	12	13	14	15	16	17	18	19	20	21	22	23	24	25	26	27	28	29	30	31	32	33	34	35	36
Run in period																																				
Recruitment																																				
Data Cleaning																																				
Final Data Cleaning																																				
Analysis and writing up																																				
Closure of dataset																																				
TSC and DMC joint meeting																																				
DMC meetings																																				
TSC meetings																																				
Collaborators meetings																																				

10.4 Trial Steering Committee

The composition and responsibilities of the trial steering committee (TSC) will comply with the PCTU SOP on Trial Oversight Committees. The role of the TSC is to provide overall supervision of the trial on behalf of the trial sponsor and trial funder to ensure trial is conducted in accordance with the principles of Good Clinical Practice (GCP) relevant regulations.

The responsibilities of the TSC will include:

- advise on the trial protocol
- advise on changes in the protocol based on considerations of feasibility and practicability
- resolve problems brought to it by the TMG
- monitor the progress of the trial, adherence to protocol and patient safety
- consider new information of relevance from other sources
- consider and act on the recommendations of the data monitoring committee (DMC) , MREC and competent authority (Medicines and Healthcare Products Regulatory Authority, MHRA) (as appropriate)
- approve trial reports and papers for publication.

The TSC will meet every 6 -12 months.

TSC members:

Independent Chair
Harold Gee MD, FRCOG
Consultant Obstetrician (retired)
0121 449 4012
0778 956 6930
harry.gee1@gmail.com

Independent Member
Ms Pollyanna Hardy
Senior Statistician, Clinical Trials Unit, National Perinatal Epidemiology Unit, University of Oxford, Old Road Campus, Headington, Oxford, OX37LF
+44 (0) 165 289 759
pollyanna.hardy@npeu.ox.ac.uk

Lay Representative
Samantha Parker
The National Childbirth Trust, Alexandra House, Oldham Terrace, Acton, London W3 6NH
+44 785 543 3002
samantha@nurturedspace.org.uk

Non Independent Members
Chief Investigator
Professor Khalid Khan
Professor of Women's Health and Clinical Epidemiology
Women's Health Research Unit, The Blizard Institute, Bart's and The London School of Medicine, Queen Mary, University of London, 58 Turner Street , Whitechapel London E1 2AB
+44 20 7882 2621
k.s.khan@qmul.ac.uk

Observers
Dr Matthew Wilson MA (Oxon) BM ChB MD FRCA
Consultant Anaesthetist
Sheffield teaching Hospitals NHS Trust
Royal Hallamshire Hospital
Glossop Road, Sheffield, S10 2JF
wilsonmja@gmail.com

Dr Philip Moore MBChB MD FRCA FFPMRCA
Consultant Anaesthetist & Honorary Senior Lecturer
Birmingham Women's NHS Foundation Trust
Mindelsohn Way, Edgbaston, Birmingham, B15 2TG
+44 121 472 1377
phil.moore@nhs.net

10.5 Consumer representation

Samantha Parker, a volunteer for The National Childbirth Trust has collaborated with the project from its inception, advised on the pilot protocol, patient information for the trial and has agreed to provide representation on the TSC.

Samantha Parker's
39 Hookstone Avenue
Harrogate
HG2 8ER
07855 433002
samantha@nurturedspace.org.uk

10.6 Data Monitoring Committee

The composition and responsibilities of the data monitoring committee (DMC) will comply with the PCTU SOP on Trial Oversight Committees. The role of the DMC is to review the accruing trial data and to assess whether there are any ethical or safety issues why the trial should not continue. A DMC independent of the trial organisers will be established and meet yearly. During recruitment, interim analyses will be supplied, in strict confidence, to the DMC, together with any other analyses the DMC may request. Other meetings of the committee may be arranged periodically, as considered appropriate by the Chair. In the light of unblinded analysis of interim data, and other evidence from relevant studies (including updated overviews of randomised controlled trials), the DMC will inform the TSC, if in their view this information provides proof beyond reasonable doubt that one or other of the treatments under investigation is either clearly indicated or contra-indicated, either for all women or for a particular subgroup of trial participants. A decision to inform the TSC will in part be based on statistical considerations. Appropriate criteria for proof beyond reasonable doubt cannot be specified precisely. A difference of at least 3 standard errors in the interim analysis of a major endpoint may be needed to justify halting, or modifying, such a trial prematurely. If this criterion were to be adopted by the DMC, it would have the practical advantage that the exact number of interim analyses would be of little importance, and so no fixed schedule is proposed. Unless modification or cessation of the protocol is recommended by the DMC, the TSC, collaborators and administrative staff (except those who supply the confidential information) will remain blind to the results of the interim analysis. Collaborators and all others associated with the trial may write through the TMG to the DMC, to draw attention to any concern they may have about the possibility of harm arising from the treatment under study. One interim analysis is planned for each year of recruitment.
DMC Members:

Independent Chair
Richard Gray
Professor of Medical Statistics
CTSU, Richard Doll Building
Old Road Campus, Roosevelt Drive, Oxford OX3 7LF
Tel: 01865 743537
Fax: 01865 743982
richard.gray@ctsu.ox.ac.uk

Independent Clinician:
Mike Grocott
Professor of Anaesthesia and Critical Care Medicine, University of Southampton
Consultant in Critical Care Medicine, University Hospitals Southampton NHS Foundation Trust
Director, NIAA Health Services Research Centre, British Oxygen Chair of Anaesthesia, Royal College of Anaesthetists
Anaesthesia and Critical Care Research Unit
CE.93 Mailpoint 24, E-Level, Centre Block
University Hospitals Southampton NHS Foundation Trust
Tremona Road, Southampton SO6 6YD
02380 795308
mike.grocott@ucl.ac.uk

Independent Clinician:
Professor Andrew Shennan
Professor of Obstetrics
Division of Women's Health, St Thomas' Hospital
10th Floor, North Wing, Westminster Bridge Road, London, SE1 7EH
020 7188 3639
andrew.shennan@kcl.ac.uk

11 Publication Policy

The Chief Investigator will co-ordinate dissemination of data from this trial. All publications using data from this trial to undertake original analyses will be submitted to the TSC for review before release. To safeguard the scientific integrity of the trial, data will not be presented in public before the main results are published without the prior consent of the TSC. The success of the trial depends on a large number of clinicians. For this reason, credit for the results will not be given to the committees or central organisers, but to all who have collaborated and participated in the trial. Acknowledgement will include all local co-ordinators and collaborators, members of the trial committees, the PCTU and trial staff. Authorship at the head of the primary results paper will be cited as a collaborative group to avoid giving undue prominence to any individual. All contributors to the trial will be listed at the end of the report, with their contribution to the trial identified. Those responsible for other publications reporting specific aspects of the trial may wish to utilise a different authorship model, such as "[name], [name] and [name] on behalf of the collaborative Group". Decisions about authorship of additional papers will be discussed and agreed by the trial investigators and the TSC. Women participating in the trial will be sent a summary of the final results of the trial, which will contain a reference to the full paper. A copy of the journal article will be available on request from the PCTU or CI.

12 References

(1) Geoghegan J, Daniels JP, Moore PA, Thompson PJ, Khan KS, Gulmezoglu AM. Cell salvage at caesarean section: the need for an evidence-based approach. BJOG 2009 May;116(6):743-7.
(2) Fong J, Gurewitsch E, Kang H, Kump L, Mack P. An analysis of transfusion practice and the role of intraoperative red blood cell salvage during cesarean delivery. Anesthesia and Analgesia 2007;104(3):666-72.
(3) Rainaldi MP, Tazzari PL, Scagliarini G, Borghi B, Conte R. Blood salvage during caesarean section. British Journal of Anaesthesia 1998;80(2):195-8.
(4) Pathak D, McDonald A, Barclay P, Mallaiah S. Use of cell salvage in caesarean section. International Journal of Obstetric Anesthesia 2010 May;19(Supplement 1, O19):S10.
(5) Arora N, Jayaraj VR, Porter M. Intraoperative cell salvage in obstetrics - observational audit of two years' practice. International Journal of Obstetric Anesthesia 2010 May;19(Supplement 1, P75):S48.
(6) Davies L, Brown TJ, Haynes S, Payne K, Elliott RA, McCollum C. Cost-effectiveness of cell salvage and alternative methods of minimising perioperative allogeneic blood transfusion: a systematic review and economic model. Health Technol Assess 2006 Nov;10(44):iii-x, 1.
(7) Carless PA, Henry DA, Moxey AJ, O'Connell D, Brown T, Fergusson DA. Cell salvage for minimising perioperative allogeneic blood transfusion. Cochrane Database Syst Rev 2010;(4):CD001888.
(8) Bland JM, Altman DG. One and two sided tests of significance. BMJ 1994 Jul 23;309(6949):248.
(9) NHS Hospitals and Science website. Price List: Blood component products / Services for 2011/2012. http://hospital blood co uk/library/pdf/Price_List_BPS_2011-12 pdf 2011 [cited 2011 Apr 26];Available from: URL: http://hospital.blood.co.uk/library/pdf/Price_List_BPS_2011-12.pdf
(10) Waters JH, Biscotti C, Potter PS, Phillipson E. Amniotic fluid removal during cell salvage in the cesarean section patient. Anesthesiology 2000;92(6):1531-6.
(11) Sullivan I, Faulds J, Ralph C. Contamination of salvaged maternal blood by amniotic fluid and fetal red cells during elective Caesarean section. British Journal of Anaesthesia 2008;101(2):225-9.
(12) National Institute for Health and Clinical Excellence. Intraoperative blood cell salvage in obstetrics - guidance. 2005.
(13) Centre for Maternal and Child Enquiries. Saving Mothers Lives: Reviewing maternal deaths to make motherhood safer: 2006-2008. BJOG: An International Journal of Obstetrics & Gynaecology 2011;118:1-203.
(14) Bodelon C, Bernabe-Ortiz A, Schiff MA, Reed SD. Factors associated with peripartum hysterectomy. Obstet Gynecol 2009 Jul;114(1):115-23.
(15) Saravanakumar K, Davies L, Lewis M, Cooper GM. High dependency care in an obstetric setting in the UK. Anaesthesia 2008 Oct;63(10):1081-6.
(16) Lataifeh I, Amarin Z, Zayed F, Al-Mehaisen L, Alchalabi H, Khader Y. Indications and outcome for obstetric patients' admission to intensive care unit: a 7-year review. J Obstet Gynaecol 2010 May;30(4):378-82.
(17) Catling S. Intraoperative cell salvage in obstetrics. Clin Risk 2008 Jan 1;14(1):14-7.
(18) Serious Hazards of Transfusion (SHOT). Annual reports and summaries. http://www shotuk org/home/ 2011 [cited 2011 Apr 26]; Available from: URL: http://www.shotuk.org/home/
(19) James AH, Patel ST, Watson W, Zaidi QR, Mangione A, Goss TF. An assessment of medical resource utilization and hospitalization cost associated with a diagnosis of anemia in women with obstetrical bleeding in the United States. J Womens Health (Larchmt) 2008 Oct;17(8):1279-84.
(20) Teig M, Harkness M, Catling S, Clarke V. Survey of cell salvage use in obstetrics in the UK. International Journal of Obstetric Anesthesia 2007;16 (Suppl 1):30.
(21) Royal College of Obstetricians and Gynaecologists. Blood Transfusions in Obstetrics (Green-top 47). 2007.

(22) Schulz KF, Altman DG, Moher D. CONSORT 2010 statement: updated guidelines for reporting parallel group randomised trials. BMJ 2010;340:c332.
(23) Boyle RK, Waters BA, O'Rourke PK. Measures of blood loss and red cell transfusion targets for caesarean delivery complicated by placenta praevia. Aust N Z J Obstet Gynaecol 2010; 50(3):242-245.
(24) Unal O, Api M, Dogance U, Balcik O, Kara O, Turan C. Do asymptomatic patients require routine hemoglobin testing following uneventful, unplanned cesarean sections? Arch Gynecol Obstet. 2010 Feb;281(2):195-9. Epub 2009 Apr 29.
(25)Westad S et al. A 12-week randomised study comparing intravenous iron sucrose versus oral ferrous sulphate for treatment of postpartum anemia Acta Obstet et Gynecol Scand, 2008; 87, 9, 916–923.
(26) Jansen AJ et al. New insights into fatigue and health-related quality of life after delivery Acta Obstet et Gynecol Scand. 2007; 86, 5, 579–584.
(27) White IR, Thompson SG. Adjusting for partially missing baseline measurements in randomized trials. Statist Med 2005; 24:993-1007].
(28) White IR, Horton NJ, Carpenter J, Pocock SJ. Strategy for intention to treat analysis in randomised trials with missing outcome data. BMJ 20011; 342:d40
(29) Hospital Episode Statistics, HES online, NHS The Information Centre for Health and Social Care, 2005-2012
http://www.hesonline.nhs.uk/Ease/servlet/ContentServer?siteID=1937&categoryID=1815
(30) Curtis L, Unit Costs of Health and Social Care, Personal Social Services Research Unit, PSSRU 2010, http://www.pssru.ac.uk/project-pages/unit-costs/2010/index.php

Appendix 1 – Communication organogram for reporting SAE's

SAE recorded on AE log and followed up until resolution

PI assesses SAE and reports to CI within **24 hours**, PI reports to local institution as per local protocol

CI reports related and unexpected SAE's to PCTU QA manager and Sponsor within **24 hours**

CI reports related and unexpected SAE's to MREC within **15 days**

CI reports to DMC every **6-12 months**

CI reports **annually** to MREC

Appendix 2 – Information with regards to Safety Reporting in Non-CTIMP Research

	Who	When	How	To Whom
**SUSAR**	Chief Investigator	Report to the Sponsor, and QA manager within 24 hours<br>MREC within 15 days of learning of the event	SAE Report form for Non-CTIMPs, available from NRES website.	Sponsor and MREC
**Urgent Safety Measures**	Chief Investigator	Contact the Sponsor and MREC Immediately<br><br>Within 3 days	By phone<br><br>Substantial amendment form giving notice in writing setting out the reasons for the urgent safety measures and the plan for future action.	Main REC and Sponsor<br><br>Main REC with a copy also sent to the sponsor. The MREC will acknowledge this within 30 days of receipt.
**Progress Reports**	Chief Investigator	Annually (starting 12 months after the date of favourable opinion)	Annual Progress Report Form (non-CTIMPs) available from the NRES website	Main REC
**Declaration of the conclusion or early termination of the study**	Chief Investigator	Within 90 days (conclusion)<br><br>Within 15 days (early termination)<br><br>*The end of study should be defined in the protocol*	End of Study Declaration form available from the NRES website	Main REC with a copy to be sent to the sponsor
**Summary of final Report**	Chief Investigator	Within one year of conclusion of the Research	No Standard Format However, the following Information should be included:-<br>Where the study has met its objectives, the main findings and arrangements for publication or dissemination including feedback to participants	Main REC with a copy to be sent to the sponsor

Appendix 3: Requirement for Blood Transfusion Flow Diagram

WHEN APPROPRIATE, AND ACCORDING TO LOTHIAN BLOOD TRANSFUSION GUIDELINES, REASSESS THIS PATIENT AFTER EACH UNIT OF BLOOD PRESCRIBED BEFORE PRESCRIBING FURTHER UNITS.

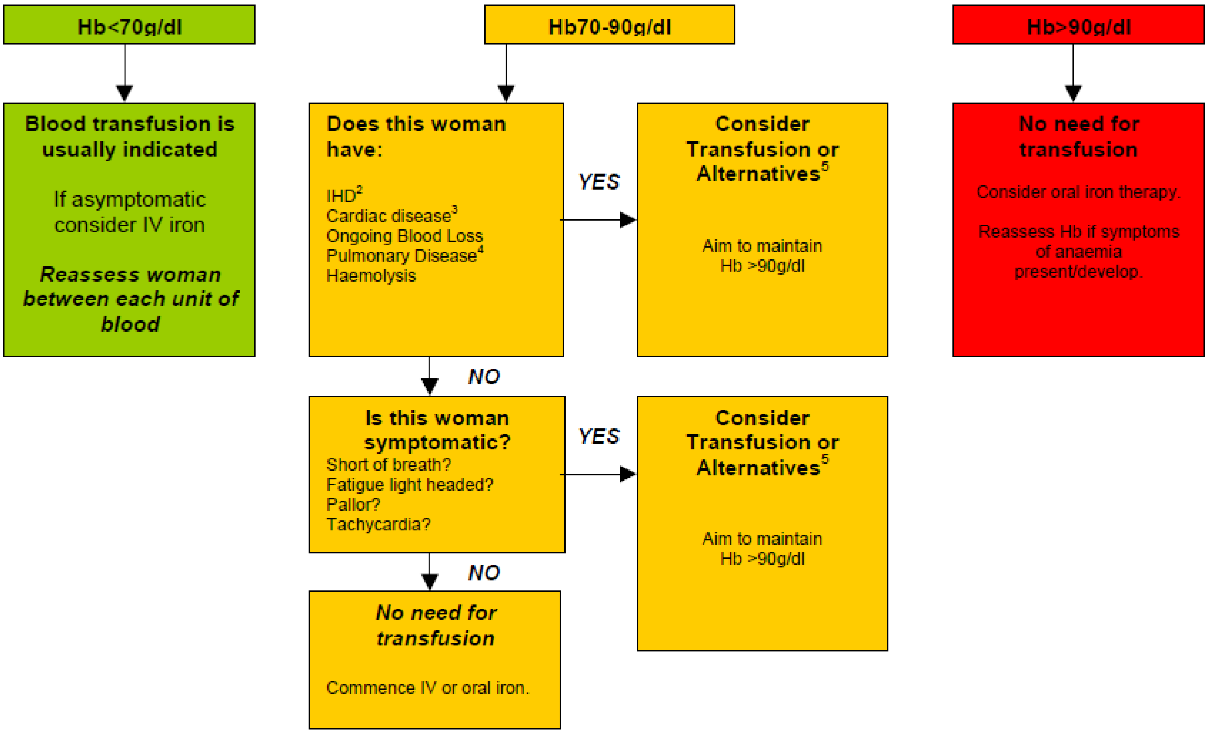

Appendix 4: Site Codes

Barts Health NHS Trust, Royal London Hospital	91
Birmingham Women's NHS Foundation Trust	92
Torbay Hospital	93
Hinchingbrooke Hospital	94
Birmingham Heartlands Hospital	96
James Cook University Hospital	97
St Michael's Hospital, Bristol	99
Royal Victoria Infirmary, Newcastle	10
Nottingham City Hospital	12
Queen's Hospital, Romford	13
Royal Hallamshire Hospital, Sheffield	14
Simpson Centre for Reproductive Health, Edinburgh	15
Singleton Hospital Swansea	16
Whipps Cross University Hospital	19
Leicester Royal Infirmary	20
Leicester General Hospital	21
Queens Medical Centre	22
Sunderland Royal Hospital	23
University Hospital of North Staffordshire	24
Royal United Hospital, Bath	25
Croydon University Hospital	27
Derriford Hospital, Plymouth	28
West Middlesex University Hospital	31
Northwick Park Hospital	32
